# Animal models in preclinical metastatic breast cancer immunotherapy research: A systematic review and meta-analysis of efficacy outcomes

**DOI:** 10.1371/journal.pone.0322876

**Published:** 2025-05-07

**Authors:** Yalda Mirzaei, Martina Hüffel, Sarah McCann, Alexandra Bannach-Brown, René H. Tolba, Julia Steitz

**Affiliations:** 1 Institute for Laboratory Animal Science, Uniklinik RWTH Aachen, Aachen, Germany; 2 Berlin Institute of Health at Charité (BIH), BIH QUEST Center for Responsible Research, Berlin, Germany; Faculty of Medicine of Tunis, TUNISIA

## Abstract

Breast cancer, particularly metastatic breast cancer (MBC), presents aggressive clinical challenges with limited treatment success. Immunotherapy has emerged as a promising approach, however, discrepancies between preclinical animal models and human cancers complicate translation to clinical outcomes. This systematic review and meta-analysis evaluated the effect of immunotherapy on primary and metastatic tumor regression in animal models of MBC and assessed the models’ appropriateness and reproducibility to improve future preclinical study design. Following a preregistered protocol in PROSPERO (CRD42021207033), we conducted searches in MEDLINE, Embase, and Web of Science databases, yielding 2255 studies for title/abstract screening and 108 studies included after full-text screening. All included studies used mouse models, assessing primary outcomes through tumor volume or weight and metastatic outcomes via nodule count or bioluminescence. Only 14% of studies fully reported experimental animal characteristics, and 43% provided detailed experimental procedures. Of 105 articles (293 comparisons) included in the meta-analysis, pooled effect sizes indicated significant reductions in both primary and metastatic tumors. However, high heterogeneity across studies and wide prediction intervals suggested substantial variability in model responses to immunotherapy. Univariable and multivariable meta-regressions failed to significantly explain this heterogeneity, suggesting additional factors may influence outcomes. Trim-and-fill and Egger’s regression tests indicated funnel plot asymmetry, implying potential publication bias and small study effects. While our analysis demonstrated positive effects of immunotherapy on MBC and highlighted variability in animal tumor models, addressing model-related heterogeneity and enhancing methodological transparency are essential to improve reproducibility and clinical translatability.

## Introduction

Breast cancer is the leading cause of cancer-related death among women worldwide, responsible for approximately 66,600 deaths globally in 2022 [1,2]. Among its subtypes, metastatic breast cancer (MBC) represents the most severe form [3]. Triple-negative breast cancer (TNBC), characterized by the absence of estrogen, progesterone, and HER2 receptors, has an aggressive clinical course, with earlier age of onset, high recurrence rates, greater metastatic potential, and shorter overall survival compared to other breast cancer subtypes [4–6]. Standard approaches to prolong survival and improve the quality of patient life include chemotherapy, radiotherapy, hormone therapy, targeted therapy, immunotherapy, and surgery [6,7]. However, MBC’s genetic heterogeneity and variable clinical behavior make it challenging to identify effective treatments [3,5,8–10]. It is well known that the immune system plays a critical role in several fundamental biological processes including the removal of cancer cells expressing tumor-specific antigens and the control of metastases. Nevertheless, cancer cells can evade immune surveillance through various mechanisms, some of which remain unidentified [11–14]. Building on that, tumor immunotherapy and targeted therapy have become therapeutic strategies to stimulate antitumor immune responses [15,16]. Despite advances, developing therapies to effectively control cancer and reduce metastasis remains an unmet need in cancer research [6,17,18]. On one hand, cancer is a complex, polygenic disease influenced by multiple environmental factors. On the other hand, discrepancies between animal models and human cancers, as well as variations in preclinical study designs impact translatability [19,20]. While new animal models can more accurately mimic human cancers by precise control over mutations [21], yet, choosing an optimal model to resemble the manifestations of MBC in humans remains challenging. Murine models particularly aid in basic research for understanding tumor biology and evaluating checkpoint inhibitors or other therapies [22–25]. Xenograft breast cancer models play a crucial role in preclinical trials, assessing drug efficacy and providing outcomes relevant to human breast cancer [19,26]. These models are used to evaluate primary tumor growth, metastatic activity, and response to therapy scenarios in both spontaneous and experimental settings [27]. Breast cancer tumors can be established in preclinical studies by choosing specific cell lines and injection sites based on tropism [28,29]. For instance, orthotopic implantation of cancer cells in the mammary fat pad creates a disease-relevant tumor microenvironment (TME) that closely resembles human breast cancer stages for better translation into the clinic [29,30]. Conversely, in subcutaneous models, the tumor cells are engrafted under the skin on the flank of the animal. Since the setup and tumor growth measurements are easier in these models, it is widely used for preclinical immunotherapies that target specific immune cell populations. However, it fails to metastasize and cannot accurately imitate the tumor microenvironment or the patient’s response in clinical studies [30–32]. Most of the complications associated with breast cancer are due to metastasis developing in regional lymph nodes or distant organs including bone, lung, liver, and brain [28,33,34]. Genetically modified and immunocompromised mouse models have long been used to investigate the progression and underlying mechanisms of breast cancer metastasis [27,28]. In these models, human breast cancer cells can be injected via various routes (subcutaneously, intravenously, intracardially, or orthotopically) with outcomes depending on the route used [27,28,35]. While tail vein injections primarily lead to lung metastasis, portal vein injection provokes liver colonization, and intracardiac infusions target multiple organs, including bone [29,36]. Over the past decade, immunotherapy has shown promise in breast cancer animal models, yet questions remain regarding the reproducibility and predictability of these models [24,25]. Thus, it is essential to examine the effects of immunotherapy in relevant preclinical models of MBC and explore sources of heterogeneity. This study aimed to systematically review published preclinical animal models of metastatic breast cancer (MBC) to evaluate the impact of immunotherapy on both primary and metastatic tumor regression compared to healthy controls through meta-analysis, as well as to investigate key study design variables essential for reproducibility in future preclinical studies.

## Materials and methods

This systematic review was conducted according to a preregistered protocol in PROSPERO (CRD42021207033; available from: https://www.crd.york.ac.uk/prospero/display_record.php?RecordID=207033) to address the research question ‘Does immunotherapy have successful effect on primary and metastatic tumor regression in the treated animal models with metastatic breast cancer, compared to control?’. The question was structured according to the PICO format with P as an animal model of metastatic breast cancer (all species), I as immunotherapy, C as untreated controls or vehicle only, and O as regression of primary and metastatic tumors (volume, area, weight). Neither risk of bias nor quality assessment were pre-planned. However, we reached a decision to assess the reporting quality based on the ARRIVE 2.0 guidelines [37,38]. There were no other deviations from the intended methodology as outlined in the protocol. The tumor model, induction method, cell line application route, drug administration route, and animal strain were analyzed as potential sources of heterogeneity.

### Search strategy

The systematic search and removal of duplicates were conducted by one author (YM). The databases MEDLINE via PubMed, Embase via embase.com, and Web of Science Core Collection via Webofscience.com RWTH Aachen university were searched to identify all potentially relevant publications until the end of February 2024. We did not examine references of included studies for additional sources. The full search strategy was based on the search components: treatment or intervention using immunotherapeutic agent, metastatic breast cancer, animal model, and tumor regression. We combined keywords and MeSH terms for PubMed and adapted this strategy to the other databases (the full search strategies are available at S1 File). The filter of “Other animals” in the PubMed database, the animal filter in Embase, and Web of Science animal related topic fields were added to the search to remove non-animal studies. Since immunotherapy is a new treatment approach for MBC, to avoid too many irrelevant basic studies, a publication date filter (Published 2010 - Feb 2024) was also applied. The returned records were transferred to EndNote version X9.3.3 (RRID: SCR_014001) to check for overlap and deduplication of our library. The final results file was then transferred to Rayyan QCRI (RRID: SCR_017584, available at http://rayyan.qcri.org) to perform the initial screening of titles and abstracts by two independent reviewers (YM and MH) with blinding of the selection/decision process. Here, we checked again the searched items for any remaining duplicates.

### Inclusion and exclusion criteria

Original controlled animal studies published in the English language using immunotherapeutic agents to achieve the main goal of treatment of metastatic breast cancer disease were included. No restrictions in intervention administration were used. Irrelevant studies were excluded based on predefined criteria. Exclusions entailed opinion articles, observational studies, case reports, reviews, clinical studies, *in vitro* studies, and treatment with nutrients or natural products. All experimental animal species of any age and sex were included in our review. Excluded populations were humans, wildlife, domestic animals, zoo animals, endangered species, and exotic animals (S2 Table). Full-text screening of the potentially eligible articles for final inclusion was subsequently carried out by the two independent reviewers (YM, MH) and articles were included if they met our pre-specified inclusion criteria. The assessment of inter-rater reliability between the two reviewers was considered according to Cohen’s kappa agreement and calculated using the Cohen’s kappa calculator tool [39,40]. Here, a minimum 0.61 agreement was required for inclusion [41]. Any disagreement over the eligibility of particular studies was resolved through discussion between the above-mentioned reviewers. A third reviewer (JS) was consulted in case the first two reviewers could not reach an agreement.

### Data extraction

One reviewer (YM) assessed each included article to extract bibliographic data (first author, year of publication, journal), data on study design (randomization, method of breast cancer induction, tumor size at the beginning time of intervention), animal model characteristics (animal species, strain, age, sex, weight, and number), intervention characteristics (type of treatment and control agents, dosage regimen and route of administration), primary outcomes: tumor weight, tumor volume, tumor size, and secondary outcomes: relative organ weights, body weight changes. The data were recorded into a purpose-built Microsoft Excel spreadsheet (RRID: SCR_016137). We first tried to extract numerical data from tables, or text. In studies where numerical data were not reported and presented only graphically in figures, a screenshot of the graphical data was taken, and adequate estimation of the outcome measurements was extracted from graphs using WebPlotDigitizer (RRID: SCR_013996). In studies where data were unobtainable, an initial request for unpublished information to the corresponding author was sent by email. If there was no response to our initial email after a minimum of seven business days, a second reminder email was sent. When there was still no response after a minimum of 30 business days following the initial email and data were missing, the results were excluded from the analyses. In cases where the outcome was measured repeatedly at different time points, only data from the endpoint (the last day of the experiment) was included. When more than one experiment was reported in the same manuscript, the experiments were only included as separate comparisons in the analyses if separate animals were used as the treatment group. If one control group was used for multiple intervention groups, the number of animals in the control group was divided by the number of intervention groups for meta-analysis. Extracted data were discussed in case of uncertainty of eligibility and final decisions were taken in a meeting with a second reviewer (JS).

### Reporting quality assessment

The reporting quality was assessed for each of the included studies based on the Animal Research: Reporting of *In Vivo* Experiments reporting guidelines (ARRIVE 2.0). Each study was assessed against the following reporting items: study design, sample size, inclusion and exclusion criteria, animal randomization to different groups, blinding, animal characteristics, experimental procedure, statistical methods used, outcome measurements, and results. To assess reporting quality, for each section mentioned in the ARRIVE Essential 10 guidelines, studies were scored as “Fully reported” if all the information for each criterion was reported, “Partially reported” if only some of the information for each criterion was reported, and “Not reported” if none of the information for each criterion was reported. Reporting quality was expressed as a summary plot using Risk-of-bias Visualization (*robvis*) tool [42].

### Data synthesis and statistical analysis

We considered meta-analyses feasible if homogeneity in the interventions, comparisons, and outcomes was present. In this manner, we divided the studies into comparisons that evaluated primary tumor regression and those that measured metastatic tumor regression. Meta-analysis was performed using R V.4.0.3 (RRID: SCR_009175) if a minimum of 4 comparisons per each primary and metastatic group were obtained. Since in all comparisons, we had a mean outcome score and a measure of its variance, we calculated a normalized difference in means, or a standardized difference in means. For primary tumor volume and tumor weight datasets, as data existed on a ratio scale and sham data could be inferred, we presented normalized mean difference [39]. This value tells us the direction and the magnitude of the treatment effect with the effect sizes typically falling between −100% and + 100%. For metastatic tumor nodules number and tumor area, we presented continuous data with the standardized mean difference (SMD) with a 95% confidence interval (CI). In studies where standard error of the mean (SEM) was provided, SD was calculated by multiplying the square root of the number of animals used in the study by the SEM value (*s.d. = s.e. × √n*). The random effects model was assigned to assess weights and the variation among studies. Additionally, we calculated the 95% prediction interval (PI) to have an estimated range that the true effect of a new study from the population of studies will fall in 95% of the cases. To have unbiased estimates of variance and covariance parameters, variance estimates were calculated using the restricted maximum likelihood (REML) approach and the degree of heterogeneity was quantified using the I^2^ statistic index. Here, based on I^2^ estimates in relation to the cumulative number of comparisons and their 95% CIs, we considered I^2^ of 50% to 70% as representing moderate heterogeneity, and I^2^ greater than 70% as a considerable heterogeneity. We performed uni- and multivariable meta-regression to explore the model-related possible sources of heterogeneity including the tumor induction method, cell line application site, animal strain, animal sex (where reported), and route of drug administration. Heterogeneity was described using Q (heterogeneity statistic), tau^2^ (estimation of between-study variance), residual I^2^ (the percentage of the residual variation that is attributable to between-study heterogeneity), and adjusted R^2^ (the proportion of between-study variance explained by the covariate). Plots were created using the forest or orchard 2.0 R package [43]. Visual inspection of funnel plots, followed by Egger’s regression test, a weighted linear regression of the treatment effect on its standard error, were used to statistically test for asymmetry arising from small study effects and possible publication bias. The ‘trim and fill’ method [44,45] was used to correct the pooled effect estimate for potential funnel plot asymmetry.

## Results

### Study selection and search results

**Fig 1:**
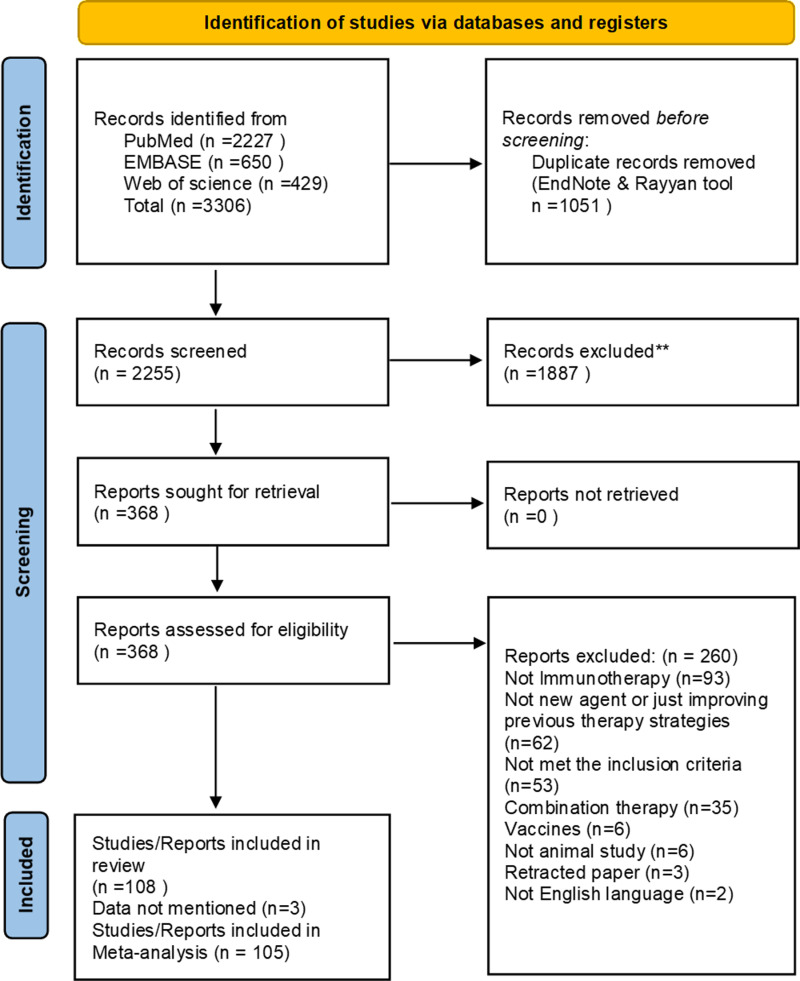
PRISMA flow chart of the study selection.

The initial search yielded in total 3306 records, of which 2227 records were from PubMed and a total of 650 articles from Embase and 429 articles from Web of Science (Fig 1). After duplicates were removed (n = 1051), there were 2255 unique studies, which were assessed for eligibility at title and abstract stage. 368 studies were included at title and abstract stage and underwent full-text analysis to judge whether they met the predefined criteria. Ultimately, 108 articles fulfilled our inclusion criteria and were included for qualitative analysis. Here, the calculated agreement based on Cohen’s kappa guidelines was 0.75 with percentage of agreement 88.88%, demonstrating a substantial agreement. Where publications included experiments on more than one model or with different treatment strategies, these were counted separately as individual comparisons. Three of these studies couldn’t be included in the quantitative meta-analysis due to incomplete data such as the animal number per group. The relevant data were extracted from the remaining 105 articles containing 293 comparisons and used for meta-analysis. In one paper (S11 File, reference number 65), we recognized a discrepancy between the outcome data mentioned in the results and the values showed in the figure. Here, based on the experiment and the likelihood of text accuracy, we decided to include outcome data mentioned in text, in our meta-analysis.

### Study characteristics

The included studies were published between 2010 and the end of February 2024 (S3 Table). All studies investigated mouse models and had ethical approval. In the majority of studies, female animals (97.2%) were used, and 3 studies reported using male animals. Out of 108 studies, 105 reported animal number per group either in the ‘methods’ or ‘results’ section but no studies discussed how the sample size was calculated. The age of the animals was reported in 91 studies (84.2%) and 15.7% of the studies reported the weight of the animals (S4 Table). In general, different methods for tumor induction were used (syngeneic, xenograft, Genetically Engineered Mouse (GEM), and patient-derived xenograft (PDX)) and animal strain and inoculation site were reported accordingly in all studies. However, 8 papers that used cell lines to induce tumors didn’t mention the injected cell number (S5 Table). Regarding the therapeutic interventions used, all studies reported the route of agent administration, and 98.1% also reported the dosage. Study duration was mentioned in all studies and varied from 14 to 105 days. However, only 103 studies reported the starting time of drug intervention either according to tumor volume at the indicated day or the day number after inoculation (S6 Table).

### Reporting quality assessment of the included studies

**Fig 2:**
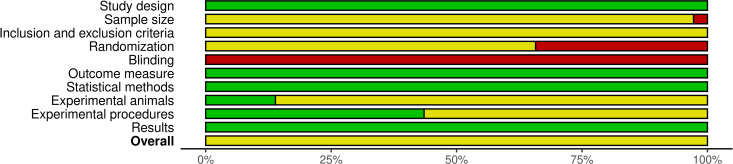
Summary plot of reporting quality assessment based on ARRIVE Essential 10. Green indicates fully reported items, Yellow indicates partially reported items, and Red indicates items not reported (generated using the *robvis* tool). Overall, the studies only partially reported the information required by the ARRIVE guidelines.

Of the 108 studies included in this SR, 73 studies (67.5%) reported randomization to control or treatment groups. However, none of these studies mentioned the method used for randomization. Moreover, measures to reduce performance bias (random housing and blinding of animal caretakers) were not reported nor was it stated whether outcome assessors were aware of the group allocation at any stage of the experiment or data analysis. Study design, outcome measures, statistical methods, and results were reported in all included studies, whereas none of them reported the method by which the sample size was chosen. Additionally, the criteria used for including and excluding animals, experimental units, or data points were partially provided. Only 14% and 43% of studies fully reported the experimental animal characteristics and experimental procedures accordingly (S7 Table). Visualization of the reporting quality scoring based on each criterion addressed in the ARRIVE Essential 10 guideline is shown in Fig 2.

### Outcome measures and exploration of heterogeneity

Primary tumor regression and metastasis tumor regression were two specific types of outcomes in the included studies and were classified and assessed separately.

#### 1. Effect of immunotherapy on primary tumor regression.

**Fig 3:**
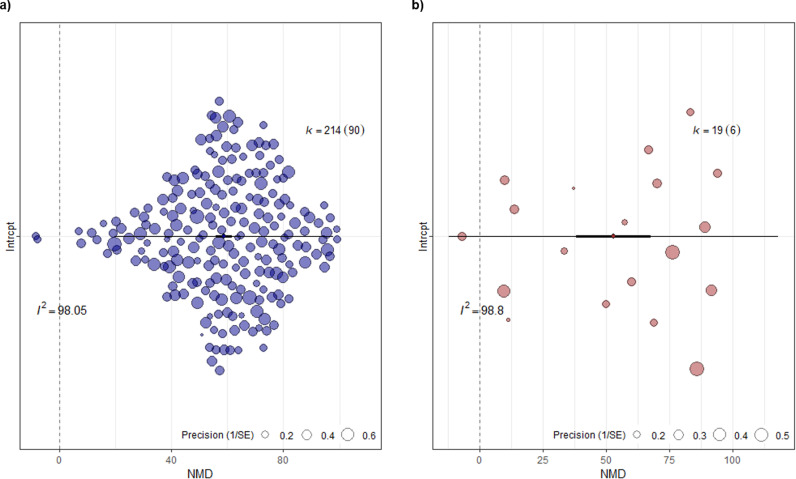
Orchard plots of meta-analysis results for primary tumor regression. a) Tumor volume, and b) Tumor weight. On each plot, the thick black line indicates 95% confidence intervals (CI), the extended black line indicates 95% prediction intervals (PI), and the midpoint is denoted by a diamond indicating the pooled normalized mean difference (NMD) effect estimate. Each circle represents a comparison and its diameter denotes the weight each comparison bears in the pooled effect size based on precision, meaning the larger the sample size the comparison has, the more precise it is and the higher its weight in the pooled effect. I^2^ reports the quantitative value for heterogeneity (98.05% and 98.8% respectively). The number of comparisons included in the meta-analysis is denoted by *k*, and with the number of unique studies denoted in brackets.

A total of 233 individual comparisons from 95 studies assessed primary tumor regression, measured either by tumor volume (n = 214) or tumor weight (n = 19). As shown in Fig 3, the pooled effect size was statistically significant, favoring immunotherapeutic agents to reduce both primary tumor volume and weight by over 50%. Specifically, there was a reduction of 58.73% (NMD, 95% CIs [55.78; 61.68], P < 0.0001, n = 214) for primary tumor volume, and 52.78% (NMD, 95% CIs [38.08; 67.47], P < 0.0001, n = 19) for primary tumor weight. However, a random-effects model showed considerable heterogeneity across the included studies for both outcomes. For tumor volume, heterogeneity was I^2^ = 98.05%, τ^2^ = 10294.25, P < 0.0001(n = 214), and for tumor weight, heterogeneity was I^2^ = 98.8%, τ^2^ = 1043.87, P < 0.0001 (n = 19). Prediction intervals further illustrated the high level of heterogeneity with a wide range positive effect sizes for primary tumor volume [19.87; 97.79]. For tumor weight, the prediction interval crossed the line of no effect, even including negative effect sizes [-12.22; 117.79]. In the tumor volume dataset (n = 214), comprising 211 comparisons in female mice and 3 in male mice, 5 experiments used genetically engineered mouse (GEM) models, 11 used patient-derived xenografts (PDX), 30 used syngeneic models, and 169 used xenografts. The most frequently used cell lines for tumor induction were MDA-MB-231 (n = 94), 4T1 (n = 22), and MCF7 (n = 22). For MDA-MB-231 and MCF7 cell lines, subcutaneous injections were more common, whereas the 4T1 cell line was more frequently injected in the mammary fat pad. In general, female *BALB/c nude* (n = 137) and *BALB/c* (n = 24) mice were the major used strains as immunosuppressant and immunocompetent mice models, respectively. In contrast, among the 19 comparisons that used primary tumor weight as the outcome, most (n = 11) used female *BALB/c* mice with 4T1 cell line injected in the mammary fat pad as an orthotopic-syngeneic model. Other cell lines used included MX1, MDA-MB-231, and MCF7.

**Fig 4:**
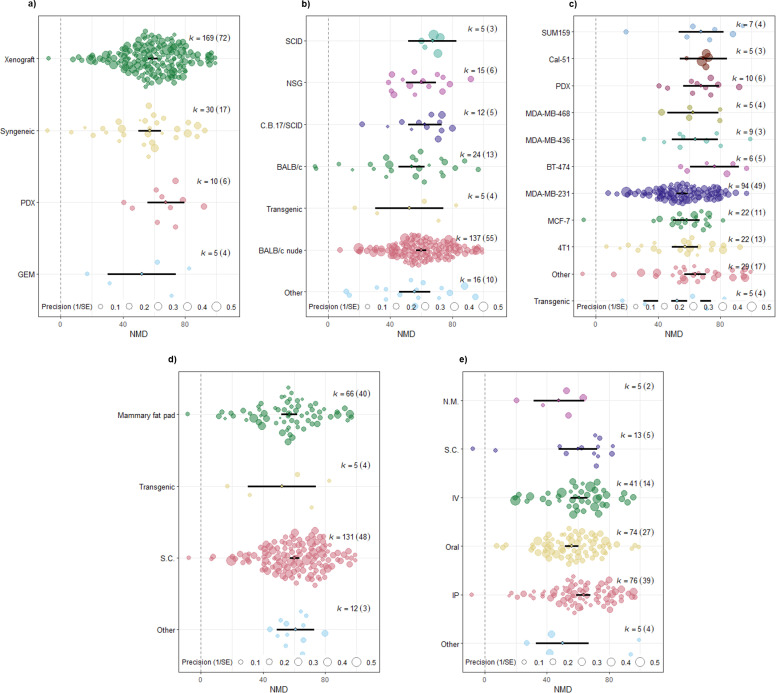
Orchard plots of univariate meta-regression analysis for primary tumor volume. a) Tumor model, b) Animal strain, c) Induction method, d) Cell line application route, and e) Drug administration route. The thick black line indicates 95% confidence intervals (CI). Each circle represents a comparison and its diameter denotes the weight each comparison bears in the pooled effect size based on precision. No difference was observed between subgroups and the p-value for test of moderators was not statistically significant (P = 0.45, P = 0.67, P = 0.10, P = 0.63, P = 0.12, respectively). The number of comparisons included in each level of the variable of interest is denoted by *k*, and with the number of unique studies denoted in brackets.

**1.1. Exploring heterogeneity in the effects of immunotherapy on tumor volume and weight:** Uni- and multivariable meta-regression analyses were conducted to examine potential sources of heterogeneity in the effects of immunotherapy on tumor volume and weight. We explored several biological and methodological factors as predictors. Specifically, we assessed whether tumor model type (syngeneic, xenograft, GEM, PDX), cell lines, cell line application route, animal sex, animal strain, and immunotherapeutic agent administration route could explain any of the observed between-study variation. In the univariable meta-regression for tumor volume outcomes, no association was found between the selected predictors and effect size, as the p-value for the test of moderators was greater than 0.05 for all predictors (Fig 4). The residual heterogeneity, or unexplained variance, remained significant, as shown by high I^2^ values across predictors: animal strain (I² = 87.07%), tumor model (I² = 87.28%), induction method (I² = 86.13%), cell line application route (I² = 87.35%), and drug administration route (I² = 86.39%), all with P < 0.0001. Univariable meta-regression based on animal sex was not conducted due to only three comparisons involving male animals, which would render the results unreliable. Additionally, we conducted a multivariable meta-regression using a mixed-effects model on the tumor volume dataset (214 comparisons), using the above predictors as moderators (S8 Table). This analysis revealed that the set of moderators (coefficients 2–24) significantly explained variation in effect sizes (test of moderators: F(df1 = 23, df2 = 190) = 1.6541, P = 0.0363). However, the amount of variance explained by these moderators was relatively low, as indicated by the test for residual heterogeneity (QE(df = 190) = 1375.8701, P < .0001, R² = 9.10%). This indicated that the moderators accounted for only a small fraction of the total variance in outcomes, suggesting that additional unmeasured factors may contribute to the observed differences between studies. Due to the limited number of unique studies (n = 6) with only 19 comparisons, meta-regression analysis was not conducted for the tumor weight dataset.

#### 2. Effect of immunotherapy on metastatic tumor regression.

**Fig 5:**
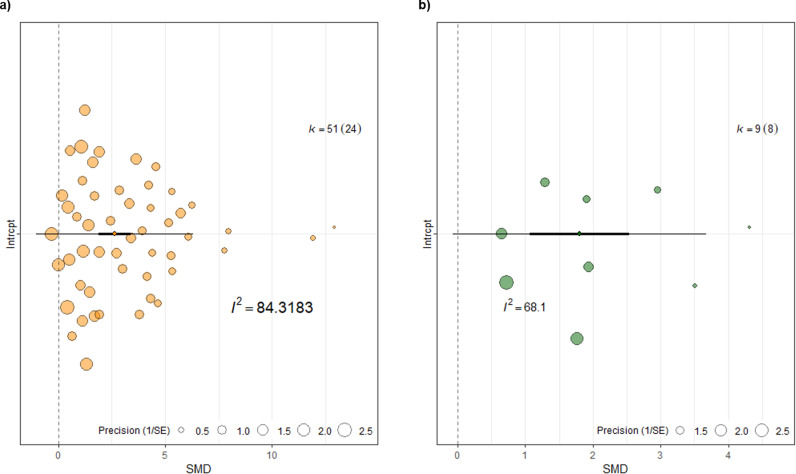
Orchard plots of metastatic tumor regression. a) Metastatic nodules number, and b) Metastatic tumor area based on bioluminescence measurement. On both plots, the thick black line indicates 95% confidence intervals (CI), the extended black line indicates 95% prediction intervals (PI), and the midpoint denoted by a diamond indicating the pooled standardized mean difference (SMD). Each circle represents a comparison and the size of it denotes the weight each comparison bears in the pooled effect size based on precision meaning the bigger sample size the comparison has, the more precise it is. I^2^ reports the quantitative value for heterogeneity (84.32% and 68.1% respectively). The number of comparisons included in the meta-analysis is denoted by *k*, and with the number of unique studies denoted in brackets.

Thirty-two studies evaluated metastatic tumor regression, and from these, 60 individual comparisons were included in the meta-analysis. Of these comparisons, 51 assessed tumor regression based on a reduction in the number of metastatic nodules in the lung, while 9 used tumor area reduction measured by bioluminescence. To provide clarity, the overall effect estimate was calculated separately for each dataset. For studies counting lung nodules, the overall effect of immunotherapeutic agents was significant (2.63 SD, 95% CIs [1.89; 3.37], P < 0.0001, n = 51), as was the effect in studies using bioluminescence (1.79 SD, 95% CIs [1.06; 2.53], P < 0.0001, n = 9), indicating a positive impact of these agents on metastasis regression. However, as shown in Fig 5, the pooled estimates indicated considerable between-study heterogeneity in both datasets. Specifically, for the lung nodule dataset, heterogeneity was Q(df = 50) = 240.8317, I² = 84.32%, P < 0.0001 (n = 51), and for the bioluminescence dataset Q(df = 8) = 24.8111, I² = 68.09%, P = 0.0017 (n = 9). The prediction intervals further highlighted this heterogeneity and spanned the line of no effect, indicating potential negative effect sizes (PIs [-1.03, 6.31] for lung nodules and PIs [-0.07, 3.66] for bioluminescence). Among the 51 comparisons counting metastatic nodules, different tumor models were employed: 3 used GEM, 1 used PDX, 27 used syngeneic, and 20 used xenograft models, all with female mice. The most commonly used cell lines were 4T1 (n = 20) and MDA-MB-231 (n = 18). For the 4T1 cell line, all studies used female *BALB/c* mice, with 17 comparisons using the mammary fat pad as the injection site, 2 using intravenous, and 1 using subcutaneous injection. On the contrary, for the MDA-MB-231 cell line, the most used route was intravenous (n = 9). Overall, female *BALB/c* and *NSG* mice were the most used strains as immunocompetent and immunosuppressant mice models, respectively. In the 9 comparisons assessing tumor area reduction by bioluminescence, the xenograft model using the MDA-MB-231-Luc cell line injected intravenously was the major tumor induction method (n = 6). Only three comparisons used the syngeneic model with the 4T1-luc cell line injected into the mammary fat pad.

**Fig 6:**
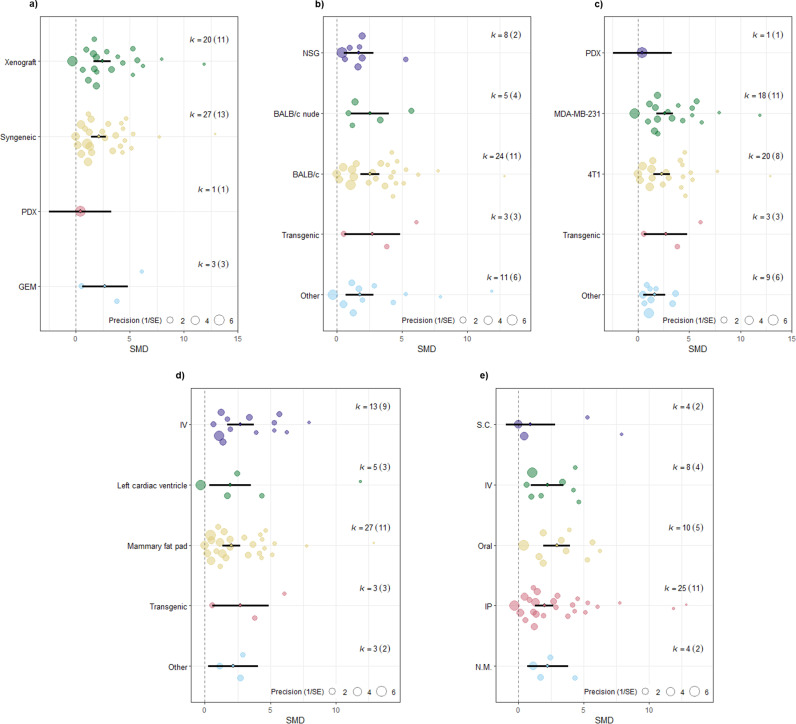
Orchard plots of univariate meta-regression analysis for metastatic nodules number. a) Tumor model, b) Animal strain, c) Induction method, d) Cell line application route, and e) Drug administration route. The thick black line indicates 95% confidence intervals (CI). Each circle represents a comparison and its diameter denotes the weight each comparison bears in the pooled effect size based on precision. No difference was observed between subgroups and the p-value for test of moderators was not statistically significant (P = 0.42, P = 0.59, P = 0.55, P = 0.81, P = 0.39, respectively). The number of comparisons included in each level of the variable of interest is denoted by *k*, and with the number of unique studies denoted in brackets.

**2.1. Exploring heterogeneity in the effect of immunotherapy on metastatic tumor regression:** We conducted univariable and multivariable meta-regression analyses to investigate potential sources of heterogeneity in the metastatic nodule reduction dataset. In the univariable meta-regression analyses, a considerable percentage of residual heterogeneity remained (Fig 6), and none of the selected predictors could explain the observed between-study differences. Specifically, residual heterogeneity values were as follows: animal strain (I² = 87.86%), tumor model (I² = 87.11%), induction method (I² = 86.67%), cell line application route (I² = 88.16%), and drug administration route (I² = 85.18%), with P < 0.0001 for all. All experiments in this dataset used female animals, so animal sex was not considered as a variable. The multivariable meta-regression also failed to account for the heterogeneity observed in the dataset. The test for residual heterogeneity remained significant QE(df = 35) = 132.7078, P < .0001, and the test of moderators (coefficients 2–16) did not reach statistical significance (F(df1 = 15, df2 = 35) = 1.3944, P = 0.2040; see S9 Table). Given the limited number of unique studies (n = 8), we decided not to not to statistically investigate the study design characteristics as sources of heterogeneity for metastatic tumor area reduction using bioluminescence measurements.

### Assessment of publication bias

**Fig 7:**
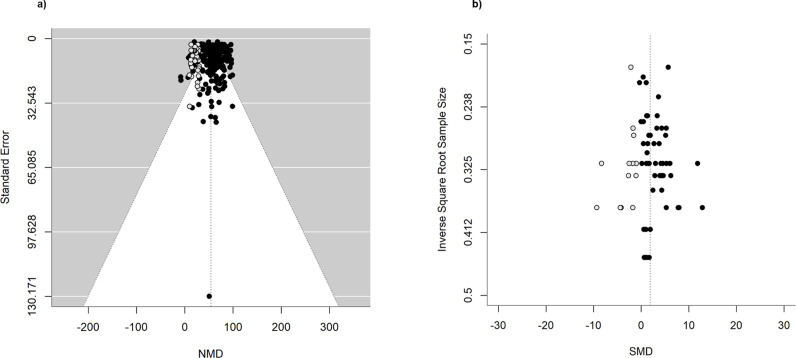
Funnel plots and Trim and Fill analysis of immunotherapy outcomes. a) Primary tumor volume, and b) Metastasis nodules number. The closed dots indicate the observed studies. The open dots indicate the missing studies imputed by the trim and fill method (n = 32 and n = 13 for a and b, respectively). The x-axis shows the observed outcome as normalized mean difference or the standardized mean difference with the y-axis representing the standard error or the inverse square root of the sample size, respectively. The two dotted lines on either side of plot (a) represent the 95% confidence intervals. The short-dash vertical line indicates the adjusted effect size including potentially missing studies under the random effects model: (a) NMD = 54.35%, and (b) SMD = 1.88 SD.

Visual inspection of the funnel plots suggested asymmetry among the larger studies for primary tumor volume. Additionally, asymmetry was observed in the funnel plot of the metastatic tumor nodules dataset, indicating the potential presence of small study effects and suggesting that some studies might be missing from our dataset, potentially biasing our findings (S10 Fig). For the primary tumor volume dataset, Egger’s regression test did not indicate significant funnel plot asymmetry (t = -0.5017, df = 212, p-val = 0.6164). However, a trim and fill analysis imputed 32 theoretically missing studies (SE = 9.5271), resulting in an adjusted effect size of 54.35% (P < 0.0001), compared to the unadjusted effect size of 58.73% NMD (Fig 7a). For the metastatic tumor nodules dataset, both the trim-and-fill analysis and Egger’s regression indicated funnel plot asymmetry and potential publication bias. The intercept of the regression model was significantly greater than zero, confirming asymmetry in the funnel plot (Egger’s test: intercept = 2.5291, t = 3.959, P = 0.000243). The trim-and-fill method further supported this by identifying statistically significant funnel plot asymmetry and suggesting the presence of potentially missing studies, which represent less effective immunotherapy experiments with lower effect sizes. The analysis estimated 13 missing studies (SE = 4.6794), resulting in an adjusted pooled effect size of 1.88 SD (P < 0.0001) compared to 2.63 SD in the original random-effects meta-analysis (Fig 7b). Although the trim-and-fill analysis adjusted the estimated effect size for both datasets, favoring immunotherapy over control, significant heterogeneity remained between studies (primary tumor volume: tau² = 392.7627, SE = 46.0535, I² = 90.34%, P < 0.0001, n = 246; metastatic nodules number: tau² = 6.0356, SE = 1.3076, I² = 89.22%, P < 0.0001, n = 64). Therefore, the adjusted intervention effect was not considered a more valid estimate of the overall effect of immunotherapy. The results should be interpreted with caution, as the inclusion of imputed missing studies likely extended the range of effect sizes and increased heterogeneity among studies. Funnel plot analysis was not conducted for the primary tumor weight or metastatic tumor area reduction datasets due to the inclusion of fewer than 10 unique studies, which would result in low statistical power to reliably detect asymmetry.

## Discussion

Breast cancer is a heterogeneous disease with variations in aggressiveness, metastasis potential, treatment response, and clinical outcomes [1,46]. While breast cancer’s causes, nature, and risk factors have been extensively studied in humans [47–53], animal models remain essential for studying tumorigenesis, testing therapeutic strategies, and monitoring drug responses [54–57]. Previous literature has evaluated a range of animal models for breast cancer, pinpointing their unique features, strengths, weaknesses, and the principles of tumor model selection to fit the purpose of the investigation [20,25,58–61]. The herein study reviewed systematically the effects of immunotherapy in published animal models of MBC and assessed the quality of reporting in these studies. Additionally, we conducted a quantitative analysis to identify potential sources of heterogeneity and explore their implications for reproducibility in future preclinical studies.

Preclinical systematic reviews and meta-analyses differ from those in the clinical field. The greater variability in experimental designs, animal models, dosing regimens, and outcome measures often introduces substantial heterogeneity [62,63]. The strength of preclinical systematic reviews lies in exploring this heterogeneity to identify factors related to study design or quality that influence outcomes. To our knowledge, this is the first systematic review and meta-analysis focused on animal models of immunotherapy in MBC.

Our assessment of reporting quality based on parameters adapted from the ARRIVE guidelines revealed significant concerns that may compromise internal validity and reproducibility. Most of the included studies did not report key information on methods to reduce biases, such as allocation, concealment, randomization, and blinding of outcome assessors. In studies where randomization was mentioned, details were insufficient for proper assessment. Although lack of reporting does not imply lack of implementation, previous systematic reviews has shown that animal experiments omitting these details tend to overestimate treatment effects [64]. Furthermore, crucial information about animal model characteristics (e.g., age, weight), experimental design (e.g., cell line, cell number, administration route), and statistical methods (e.g., power calculations, sample size justification) was often missing. These findings are in line with previous studies that evaluate the internal validity of preclinical experiments [65–68]. Though we did not address external validity (e.g., representativeness of animal samples, clinical relevance of models, and animal–human species differences), it is essential to ensure internal validity and reproducibility to enhance the clinical translatability of preclinical findings [68,69]. Therefore, improving transparency and methodological rigor in reporting is vital for increasing the reproducibility and reliability of *in vivo* tumor models across research laboratories and achieving more translatable results for clinical trials.

A key finding of our study was that most immunotherapy studies in MBC models used xenografts in immunodeficient mice, particularly nude mice. Given their lack of functional T cells, these models are suboptimal for evaluating immune-based therapies, as they fail to replicate tumor–immune system interactions [70]. While xenograft models offer advantages in tumor engraftment and reproducibility, their limitations should be considered when interpreting immunotherapy outcomes [71]. Syngeneic and genetically engineered models with intact immune systems provide more physiologically relevant platforms for assessing immunotherapies [72].

For the quantitative analysis, studies were divided into primary and metastatic tumor regression categories to account for inter-tumoral heterogeneity and diversity between primary and metastasis tumors [73]. Studies measured tumor regression by weight or volume for primary tumors and by nodule count or area for metastatic tumors. Results indicated that immunotherapy had a significant, positive effect, with a reduction of over 50% in primary tumor volume and weight, as well as a reduction in metastatic tumor nodules number and area. However, statistical analyses revealed moderate to considerable heterogeneity, suggesting substantial variability in immunotherapy effectiveness across studies. Prediction intervals further showed high heterogeneity, with ranges that included negative effect sizes for primary tumor weight and both metastatic tumor datasets. This estimation is based on the effect sizes observed in the meta-analysis and broadly corresponds to their range. The heterogeneity likely reflects variability in study methodologies, such as randomization and selection processes, which may introduce potential selection bias. Consequently, the prediction interval should be interpreted as describing the range of observed effect sizes, rather than predicting the range of effect sizes expected in future studies [74].

We were particularly interested in whether characteristics related to the different tumor models used in the included studies might help to explain the observed heterogeneity. The diversity of tumor models in our review arises from the use of both transplanted and genetically engineered models. Additionally, factors such as the origin and type of tumor tissue or cells (animal or human), their quantity, and the site of implantation contribute to biological heterogeneity [25]. We examined model-related sources of heterogeneity through univariate and multivariable meta-regression analyses, but much of the variability remained unexplained, underscoring the need for further evaluation. Our data show that MDA-MB-231, MCF7, and 4T1 cell lines, typically administered via the mammary fat pad or subcutaneously, dominated primary tumor studies. In studies on metastatic tumor regression, the MDA-MB-231 and 4T1 cell lines were the most commonly used, with administration via the mammary fat pad or intravenous route.

### Limitations

This systematic review and meta-analysis provided valuable insights into the effects of immunotherapy on MBC regression and an overview of the animal models used. However, our analysis has limitations. First, factors such as the injected cell number, cell environment (e.g., media or Matrigel®), tumor size at treatment onset, and the type and dosage of immunotherapeutic agent are known to influence tumor establishment and antitumor responses. However, limited data on these factors prevented a post-hoc analysis with a sufficient sample size to fully evaluate their impact on the unexplained heterogeneity in our analyses. Second, small study effects and possible publication bias may influence our findings, as larger studies with significant results are more likely to be published [66,68,75]. The absence of neutral or negative results hinders the development of new models for drug testing, as it increases the risk of duplicating past failures. Additionally, it impedes efforts to identify optimal animal models, leading to unnecessary use of additional animals. Third, our analysis relied on the trim-and-fill method to assess publication bias, which assumes that funnel plot asymmetry is solely indicative of publication bias. While reliance on publicly available information, selective reporting of outcomes and analyses, and the absence of reports on unsuccessful interventions contribute to potential publication bias, observed heterogeneity and publication bias may also interact and influence each other. Publication bias can significantly impact heterogeneity estimates [76,77], and moderate to high heterogeneity can reduce the power of the trim-and-fill method [78,79]. As a result, our analysis was affected by both observed heterogeneity and potential publication bias. Moreover, the lack of uniformity and standardized protocols in preclinical studies makes it challenging to make generalized conclusions Therefore, findings from preclinical meta-analyses should be interpreted within this context.

## Conclusions

Overall, this systematic review and meta-analysis highlighted the wide variety of tumor models used to investigate immunotherapeutic agents in MBC. Our findings underscored several factors that contribute to the translational gap and lack of reproducibility in these models. While our results showed that immunotherapy had significantly beneficial pooled effects on both primary and metastasis tumor regression in MBC, improved adherence to ARRIVE guidelines in reporting animal studies is needed. Moreover, our work encourages further systematic reviews on animal models used for other therapies across different cancer types. Collecting and organizing this data could support the development of an open-access database of animal models categorized by intervention and degree of tumor regression, allowing researchers to select the most suitable model for their study goals before initiating an animal experiment.

## Supporting information

S1 File Search strategy. (DOCX)

S2 Table Summary of study inclusion and exclusion criteria. (DOCX)

S3 Table Main bibliographic data of the 100 studies included in Systematic review. (DOCX)

S4 Table Main animal characteristics of the 100 studies included in Systematic review. (DOCX)

S5 Table Main study design characteristics of the 100 studies included in Systematic review. (DOCX)

S6 Table Main experiment design characteristics of the 100 studies included in Systematic review. (DOCX)

S7 Table Reporting quality assessment using the ARRIVE guidelines. (DOCX)

S8 Table Multivariable meta-regression analyses for primary tumor volume regression dataset. (DOCX)

S9 Table Multivariable meta-regression analyses for metastatic lung nodules number dataset. (DOCX)

S10 Fig Funnel plots of immunotherapy outcomes. (DOCX)

S11 File List of the included studies in Systematic review and Meta-analyses and PRISMA 2020 Checklist. (DOCX)

S12 File All studies identified in the literature search, including the reason(s) for exclusion. (XLSX)

S13 File All data extracted from the primary research sources for the systematic review and meta-analysis. (XLSX)
