# Supplementary material for: Animal models in preclinical metastatic breast cancer immunotherapy research: A systematic review and meta-analysis of efficacy outcomes
Source: PLoS One. 2025 May 7;20(5):e0322876. doi: 10.1371/journal.pone.0322876 (PMC12057864; doi:10.1371/journal.pone.0322876)
Supplement: S1 File — (DOCX) [file pone.0322876.s001.docx]

**S1 File. Search strategy**

1. ***Pubmed***

((((treatment[Title/Abstract] OR therapy[Title/Abstract] OR intervention[Title/Abstract] OR evaluation[Title/Abstract] OR destroy[Title/Abstract] OR stop*[Title/Abstract] OR boost*[Title/Abstract] OR stimulate*[Title/Abstract] OR promote*[Title/Abstract] OR decrease*[Title/Abstract] OR increase*[Title/Abstract] OR affect*[Title/Abstract] OR effect*[Title/Abstract] OR prevent*[Title/Abstract] OR prevention[Title/Abstract] OR inhibit*OR attenuate*[Title/Abstract] OR enhance*[Title/Abstract] AND (breast cancer[MeSH Terms])) OR (metastatic breast cancer[Title/Abstract] OR metastatic breast tumor*[Title/Abstract] OR metastatic breast cancer cell*[Title/Abstract] OR metastatic breast tumor cell*OR metastatic mammary cancer[Title/Abstract] OR metastatic mammary tumor*[Title/Abstract] OR metastatic mammary cancer cell*[Title/Abstract] OR metastatic mammary tumor cell*[Title/Abstract]) AND (targeted therapy[Title/Abstract] OR target therapy[Title/Abstract] OR immune therapy[Title/Abstract] OR immunotherapy[Title/Abstract] OR immune oncology[Title/Abstract] OR antibody*[Title/Abstract] OR monoclonal antibody*[Title/Abstract] OR small molecule*[Title/Abstract] OR peptide*[Title/Abstract] OR nano-particle*[Title/Abstract] OR biomarker*[Title/Abstract] OR marker*[Title/Abstract] OR inhibitor*[Title/Abstract] OR receptor*[Title/Abstract] OR antigen receptor*[Title/Abstract] OR therapeutic antibody*[Title/Abstract] OR checkpoint inhibitor*[Title/Abstract] OR immune stimulator*[Title/Abstract] OR stimulator*[Title/Abstract] OR immune stimulation*[Title/Abstract] OR t-cell*[Title/Abstract] OR modulator*[Title/Abstract] OR immune system modulator* CAR t-cell[Title/Abstract] OR cytokine[Title/Abstract] OR molecular target*[Title/Abstract] OR molecular targeted[Title/Abstract])) AND (animal*[Title/Abstract] OR animal study*[Title/Abstract] OR in vivo[Title/Abstract] OR in vivo study*[Title/Abstract] OR animal model*OR in vivo model[Title/Abstract])) NOT (in vitro[Title/Abstract])) NOT (review[Title/Abstract]) Filters: English, Other Animals, from 2010/1/1 - 2024/3/1

1. ***Web of science:***

(((((TS=(treatment OR therapy OR intervention OR evaluation OR destroy OR stop*OR boost* OR stimulate* OR promote* OR decrease* OR increase* OR affect* OR effect* OR prevent* OR inhibit* OR attenuate* OR enhance*)) AND TI=( metastatic breast neoplasms OR metastatic breast cancer OR metastatic breast tumor*OR metastatic breast cancer cell* OR metastatic breast tumor cell* OR metastatic mammary cancer OR metastatic mammary tumor* OR metastatic mammary cancer cell* OR metastatic mammary tumor cell*)) AND TS=(targeted therapy OR target therapy OR immune therapy OR immunotherapy OR immune oncology OR antibody* OR monoclonal antibody* OR small molecule* OR peptide* OR nano-particle* OR biomarker* OR marker* OR inhibitor* OR receptor* OR antigen receptor* OR therapeutic antibody* OR checkpoint inhibitor* OR immune stimulator* OR stimulator* OR immune stimulation* OR t-cell* OR modulator*OR immune system modulator* CAR t-cell OR cytokine or molecular target* OR molecular targeted)) AND TS=(animal* OR animal study* OR in vivo OR in vivo study* OR animal model* OR in vivo model*)) NOT TI=(in vitro)) AND ((PY==("2024" OR "2023" OR "2022" OR "2021" OR "2020" OR "2019" OR "2018" OR "2017" OR "2016" OR "2015" OR "2014" OR "2013" OR "2012" OR "2011" OR "2010") AND LA==("ENGLISH") AND DT==("ARTICLE")) NOT (DT==("REVIEW")))

1. ***EMBASE***

('breast neoplasms':ti,ab,kw OR 'metastatic breast cancer':ti,ab,kw OR 'metastatic breast tumor*or metastatic breast cancer cell*':ti,ab,kw OR 'metastatic breast tumor cell*':ti,ab,kw OR 'metastatic mammary cancer':ti,ab,kw OR 'metastatic mammary tumor*':ti,ab,kw OR 'metastatic mammary cancer cell*':ti,ab,kw OR 'metastatic mammary tumor cell*':ti,ab,kw) AND ('targeted therapy':ti,ab,kw OR 'target therapy':ti,ab,kw OR 'immune therapy':ti,ab,kw OR immunotherapy:ti,ab,kw OR 'immune oncology':ti,ab,kw OR antibody*:ti,ab,kw OR 'monoclonal antibody*':ti,ab,kw OR 'small molecule*':ti,ab,kw OR peptide*:ti,ab,kw OR 'nano particle*':ti,ab,kw OR biomarker*:ti,ab,kw OR marker*:ti,ab,kw OR inhibitor*:ti,ab,kw OR receptor*:ti,ab,kw OR 'antigen receptor*':ti,ab,kw OR 'therapeutic antibody*':ti,ab,kw OR 'checkpoint inhibitor*':ti,ab,kw OR 'immune stimulator*':ti,ab,kw OR stimulator*:ti,ab,kw OR 'immune stimulation*':ti,ab,kw OR 't cell*':ti,ab,kw OR 'modulator*or immune system modulator* car t-cell':ti,ab,kw OR cytokine:ti,ab,kw OR 'molecular target*':ti,ab,kw OR 'molecular targeted':ti,ab,kw) AND (treatment:ti,ab,kw OR therapy:ti,ab,kw OR intervention:ti,ab,kw OR evaluation:ti,ab,kw OR destroy:ti,ab,kw OR 'stop*or boost*':ti,ab,kw OR stimulate*:ti,ab,kw OR promote*:ti,ab,kw OR decrease*:ti,ab,kw OR increase*:ti,ab,kw OR affect*:ti,ab,kw OR effect*:ti,ab,kw OR prevent*:ti,ab,kw OR inhibit*:ti,ab,kw OR attenuate*:ti,ab,kw OR enhance*:ti,ab,kw) NOT 'in vitro study':ti AND (animal*:ti,ab,kw OR 'animal study*':ti,ab,kw OR 'in vivo':ti,ab,kw OR 'in vivo study*':ti,ab,kw OR 'animal model*':ti,ab,kw OR 'in vivo model*':ti,ab,kw) AND [2010-2024]/py AND 'article'/it
