# Supplementary material for: Animal models in preclinical metastatic breast cancer immunotherapy research: A systematic review and meta-analysis of efficacy outcomes
Source: PLoS One. 2025 May 7;20(5):e0322876. doi: 10.1371/journal.pone.0322876 (PMC12057864; doi:10.1371/journal.pone.0322876)
Supplement: S2 Table — (DOCX) [file pone.0322876.s002.docx]

**S2 Table. Summary of study inclusion and exclusion criteria**

|  | **Inclusion Criteria** | **Exclusion Criteria** |
| --- | --- | --- |
| **Type of studies** | - Original controlled animal studies with a separate control group. - Only English publications available in full text | - Case studies, opinion studies, observational studies, case reports, reviews, abstracts without adequate data, cross-over studies - Studies without a separate control group - Studies if the main goal as treating metastatic breast cancer using immunotherapy agents is not addressed. |
| **Type of participants** | - All experimental animal species of any age and sex | - Human studies (Clinical trials) - *In vitro* studies - Wild life, domestic animals, zoo animals, endangered species and exotic animals |
| **Intervention** | - All animal studies performed to achieve the main goal of treatment of metastatic breast cancer using immunotherapy | - Treatment with nutrients or natural products - Surgery, Chemotherapy, Hormone therapy, Gene therapy, Photo & thermo therapy, Cytotoxic therapy, Radio therapy - Combination therapy, Vaccines, Combination of immunotherapy and chemotherapy, Antibody drug conjugates (ADC), Co-targeted therapy |
| **Comparator** | - Control group receiving no treatment or vehicle control (negative control group) | - Not a separate control group |
