# Supplementary material for: Animal models in preclinical metastatic breast cancer immunotherapy research: A systematic review and meta-analysis of efficacy outcomes
Source: PLoS One. 2025 May 7;20(5):e0322876. doi: 10.1371/journal.pone.0322876 (PMC12057864; doi:10.1371/journal.pone.0322876)
Supplement: S3 Table — (DOCX) [file pone.0322876.s003.docx]

**S3 Table. Main bibliographic data of the 100 studies included in Systematic review**

| Reference | First Author | Journal | Year |
| --- | --- | --- | --- |
| (1) | Kenatora Yamanaka | International Journal of Oncology | 2011 |
| (2) | Chul-Yong Park | European Journal of Cancer | 2011 |
| (3) | Swati Biswas | PLoS ONE | 2011 |
| (4) | David Marion | International Journal of Oncology | 2012 |
| (5) | Yesim Gökmen-Polar | Breast Cancer Research Treatment | 2012 |
| (6) | Jinpiao Lin | Cancer ImmuNMlogy, ImmuNOTherapy | 2012 |
| (7) | Ji Hye Park | Invest New Drugs | 2012 |
| (8) | Chandra R Tate | Breast Cancer Research | 2012 |
| (9) | Vita Golubovskaya | Anti-Cancer Agents in Medicinal Chemistry | 2013 |
| (10) | Megumi Kawamoto | Molecular Cancer Therapeutics | 2013 |
| (11) | Zhong Hua li | Oncology Reports | 2013 |
| (12) | Timmy Mani | Bioorganic & Medicinal Chemistry | 2013 |
| (13) | Xiaoyi Liu | Cellular ImmuNMlogy | 2013 |
| (14) | Xinrong Ma | OncoImmuNMlogy | 2013 |
| (15) | Thaiz Ferraz Borin | PLoS ONE | 2014 |
| (16) | Mahnaz Janghorban | PNAS | 2014 |
| (17) | Esak Lee | Scientific Reports | 2014 |
| (18) | Ju-Hee Lee | Oncotarget | 2014 |
| (19) | David A. Proia | Clinical Cancer Research | 2014 |
| (20) | Lyndsay V. Rhodes | Breast Cancer Research Treatment | 2014 |
| (21) | Ji Yeon Son | Molecular Cancer Therapeutics | 2014 |
| (22) | Marc Turini | Oncotarget | 2014 |
| (23) | Wei Wang | Nature Communications | 2014 |
| (24) | Hiromi Hiyoshi | Scientific Reports | 2014 |
| (25) | Li Liu | Cellular Physiology and Biochemistry | 2014 |
| (26) | Chandra Bartholomeusz | Molecular Cancer Therapeutics | 2015 |
| (27) | María Dolores Cuenca-López | Oncotarget | 2015 |
| (28) | Ai-ling liang | Drug Design, Development and Therapy (Dove press) | 2015 |
| (29) | Sheng-Bin Peng | Molecular Cancer Therapeutics | 2015 |
| (30) | Sheetal Pundir | Cancer Research | 2015 |
| (31) | Xianzhou Song | PLoS ONE | 2015 |
| (32) | Shaohua Wie | Molecular Medicine Reports | 2015 |
| (33) | Xiao Yu Wu | Oncotarget | 2015 |
| (34) | Yao Yu | Biochemical and Biophysical Research Communications | 2015 |
| (35) | Amelie Fouque | Journal of Medicinal Chemistry | 2015 |
| (36) | Nagore I. Marín-Ramos | Journal of Medicinal Chemistry | 2015 |
| (37) | Xiu-Rong Ren | Bresat Cancer Research | 2015 |
| (38) | Alexia Arpel | Oncotarget | 2016 |
| (39) | Wells S. Brown | Molecular Cancer Therapeutics | 2016 |
| (40) | Jinxu Fang | International Journal of Cancer | 2016 |
| (41) | Joon-Suk Park | Oncotarget | 2016 |
| (42) | Chloé Prunier | Cancer Research | 2016 |
| (43) | Jiang-Jiang Qin | Oncotarget | 2016 |
| (44) | Fei Shen | Molecular Cancer Research | 2016 |
| (45) | Jianbo Zhang | Oncotarget | 2016 |
| (46) | Zhuobin Xu | Cancer Letters | 2016 |
| (47) | Qin Zhou | Biochemical Pharmacology | 2016 |
| (48) | Michael J. Gray | Breast Cancer Research | 2016 |
| (49) | Wen-hua Chen | Acta Pharmacologica Sinica | 2017 |
| (50) | Herryawan Ryadi Eziwar Dyari | The Faseb Journal | 2017 |
| (51) | Tessa Humphries-Bickley | Molecular Cancer Therapeutics | 2017 |
| (52) | Appu Rathinavelu | Tumor Biology | 2017 |
| (53) | Ping Zhong | International Journal of NaNMmedicine | 2017 |
| (54) | Xin Li | European Journal of Pharmaceutical Sciences | 2017 |
| (55) | Yasuro Sugimoto | Bioorganic & Medicinal Chemistry | 2017 |
| (56) | D. Torres-García | Vaccine | 2017 |
| (57) | Charli Dominguez | JCI Insight | 2017 |
| (58) | Hassan Y. Ebrahim | Journal of Natural Products | 2017 |
| (59) | Huber-RuaNM | Annals of Oncology | 2017 |
| (60) | Arthee Jahangir | OncoImmuNMlogy | 2017 |
| (61) | Jennifer R. Riggs | Journal of Medicinal Chemistry | 2017 |
| (62) | Wenying Yu | Journal of Medicinal Chemistry | 2017 |
| (63) | Heba Alshaker | Breast Cancer Research and Treatment | 2018 |
| (64) | Jae-Jin Lee | Scientific Reports | 2018 |
| *(65) | Qian Lei | Cell Death & Disease | 2018 |
| (66) | Yan Li | Molecular Medicine Report | 2018 |
| (67) | Banghua Wang | Journal of Breast Cancer | 2018 |
| (68) | Dan Zhu | Molecular Cancer Therapeutics | 2018 |
| (69) | Yue Chen | Journal of Drug Targeting | 2018 |
| (70) | Benjamin B. Kasten | International Journal of Molecular Science | 2018 |
| (71) | Elena Mariotto | Cancers | 2018 |
| (72) | Anna Capasso | Molecular Cancer Therapeutics | 2019 |
| (73) | Lei Di | Journal of Experimental & Clinical Cancer Research | 2019 |
| (74) | Denise Garcia | Annals of Surgical Oncology | 2019 |
| (75) | Jangsoon Lee | Breast Cancer Research and Treatment | 2019 |
| (76) | In Hae park | Scientific Reports | 2019 |
| (77) | Yingying Shen | International Journal of Cancer | 2019 |
| (78) | Jilai Tian | Breast Cancer Research and Treatment | 2019 |
| (79) | Suryavathi Viswanadhapalli | Molecular Cancer Therapeutics | 2019 |
| (80) | Zhuan Zhou | EBioMedicine | 2019 |
| (81) | Tae-Min Cho | Cancer Letters | 2019 |
| (82) | Mouhamed Idrissou | OMICS | 2019 |
| (83) | Junya Kawai | Journal of Medicinal Chemistry | 2019 |
| (84) | Ki Yeon Kim | Journal of Experimental & Clinical Cancer Research | 2019 |
| (85) | Zheng Qiao | International Journal of Molecular Sciences | 2019 |
| (86) | H. Xiao | Neoplasma | 2019 |
| (87) | Shanshan Deng | Molecular Cancer Therapeutics | 2020 |
| (88) | Ping Hu | Journal of Materials Chemistry B | 2020 |
| (89) | Sean P. Kennedy | Oncogene | 2020 |
| (90) | Li Pan | Breast Cancer Research and Treatment | 2020 |
| (91) | Lan Lin | International ImmuNMpharmacology | 2020 |
| (92) | Jian-Hua Liu | Experimental Cell Research | 2020 |
| (93) | Rui Wu | European Journal of Medicinal Chemistry | 2020 |
| (94) | Jing Chen | Life Sciences | 2020 |
| (95) | Laura Andrini | Folia Medica | 2020 |
| (96) | Qianming Li | Experimental Cell Research | 2020 |
| (97) | Niaz Mahmood | Bone Research | 2020 |
| (98) | Soo Kyung Park | Biochemical Pharmacology | 2020 |
| (99) | Arjanneke F. van de Merbel | International Journal of Molecular Science | 2021 |
| (100)  (101)  (102)  (103)  (104)  (105)  (106)  (107)  (108) | Rongyn Zhao  Yang Li  Ming Yi  Yuzhu Zhang  Xiaoyong Dai  Guiping Gao  Nilufar Ali  Penelope Desroys du Roure  Liang Lyu | European Journal of Medicinal Chemistry  Journal of Medicinal Chemistry  Journal for ImmunoTherapy of Cancer  Cancer Letters  European Journal of Pharmacology  European Journal of Medicinal Chemistry  Biomed Pharmacother.  Journal for ImmunoTherapy of Cancer  British Journal of Cancer | 2021  2021  2022  2023  2023  2023  2024  2024  2024 |

*** In this paper, we recognized a discrepancy between the numbers mentioned in result part and the values showed in figure. Data mentioned in the result part of manuscript was included in meta-analysis.**
