# Supplementary material for: Animal models in preclinical metastatic breast cancer immunotherapy research: A systematic review and meta-analysis of efficacy outcomes
Source: PLoS One. 2025 May 7;20(5):e0322876. doi: 10.1371/journal.pone.0322876 (PMC12057864; doi:10.1371/journal.pone.0322876)
Supplement: S4 Table — (DOCX) [file pone.0322876.s004.docx]

**S4 Table. Main animal characteristics of the 100 studies included in Systematic review**

| Article | Animal strain | Sex | Age | Weight | Housing type | Randomization | |
| --- | --- | --- | --- | --- | --- | --- | --- |
|  |  |  |  |  |  |  | |
| 1  2  3  4  5  6  7  8  9  10  11  12  13  14  15  16  17  18  19  20  21  22  23  24  25  26  27  28  29  30  31  32  33  34  35  36  37  38  39  40  41  42  43  44  45  46  47  48  49  50  51  52  53  54  55  56  57  58  59  60  61  62  63  64  65  66  67  68  69  70  71  72  73  74  75  76  77  78  79  80  81  82  83  84  85  86  87  88  89  90  91  92  93  94  95  96  97  98  99  100  101  102  103  104  105  106  107  108 | BALB/c nude & C.B.17/SCID  MMTV/c-Neu Transgenic & BALB/c  BALB/c & Athymic nude  BALB/c & Athymic nude  BALB/c  Athymic nude  Athymic nude  C.B.17/SCID  Athymic nude  Athymic nude  BALB/c nude  NMD/SCID  TA2 transgenic  BALB/c  Athymic nude  NSG  Athymic nude  BALB/c nude  CB.17/SCID & BALB/c  SCID/beige  MMTV/c-Neu Transgenic & BALB/c  NMRI nude  Athymic nude  BALB/c nude  BALB/c mice  Athymic nude  BALB/c nude  Athymic nude  SCID  Athymic nude  Athymic nude  BALB/c nude  BALB/c nude  BALB/c nude  NSG  Athymic nude  SCID  Athymice nude  NSG & BALB/c  BALB/c  BALB/c nude  NMRI nude  Athymic nude  C.B 17 SCID  BALB/c nude  BALB/c nude  BALB/c nude  C57BL/6 & Balb/c  BALB/c nude  BALB/c nude  Athymic nude  NCr nude  Athymic nude  BALB/c nude  Athymic nude  BALB/c  C.B-17 SCID  Athymic nude  BALB/c  BALB/c  SCID  BALB/c nude  BALB/c nude  NMD/SCID  BALB/c nude  BALB/c nude  BALB/c nude  NSG/CB17 SCID  BAlB/c nude  Athymic nude  C57BL/6 & NMD/SCID  Athymic nude  BALB/c nude & MMTV-PyMT Transgenic  Athymic nude  NSG  BALB/c nude  NSG  Athymic nude  Athymic nude & SCID  Athymic nude  BALB/c  BALB/c nude  BALB/c nude  MMTV-PyMT Transgenic  BALB/c  BALB/c  NSG  BALB/c  BALB/c nude  BALB/c  BALB/c  BALB/c nude  BALB/c nude  BALB/c nude  C3H/S-strain  MMTV-PyMT Transgenic& Athymic nude  CD-1 nude  BALB/c nude  BALB/c nude  BALB/c nude  BALB/c nude & BALB/c  BALB/c mice  BALB/c nude  BALB/c nude  Athymic BALB/c  BALB/c  Athymic & Swiss nude (NU(Ico)-Foxn1nu)  BALB/c nude | F  F  F  F  F  F  F  F  F  F  F  F  F  F  F  F  F  F  F  F  F  F  F  F  F  F  F  F  F  F  F  M  F  F  F  F  F  F  F  F  F  F  F  F  F  F  F  F  F  F  F  F  F  F  F  F  F  F  F  F  F  F  F  F  F  F  M  F  F  F  F  F  F  F  F  F  F  F  F  F  F  F  F  F  F  F  F  F  F  F  F  F  F  F  M  F  F  F  F  F  F  F  F  F  F  F  F  F | 5w & 9w  8w &10w  4-5w  6w  *NM  5w  6w  4-6w  6w  7w  4-6w  NM  48W  NM  6-8w  NM  4-5w  6w  7-12& 8w  4-6w  32w& NM  NM  4-6w  4-5w  7w  6-8w  5w  4-5w  NM  5w  4-5w  4w  NM  NM  7w  6w  NM  8w  4w& 4w  NM  6w  NM  4-6w  6W  NM  6w  8-10w  4-6w& 6w  4-5w  6w  4-5w  8-9w  5-6w  6-7w  NM  4-6w  6w  5-6w  NM  12w  NM  6w  6-8w  5w  6w  6W  5w  4-8w  8-10w  5-10w  7w& 7w  5-6w  6w& 8w  5-6w  4-6w  6-7w  4-8w  6w  8w  8w  6w  6w  5w  9w  8w  6-7w  5-8w  NM  6-8w  6w  6-8w  5w  NM  6-8w  9w  9w& 6w  4-6w  4-5w  5w  6-8 w  6-8 w  NM  4-6w  6w  4-6w  8w  6w & 5w  36w | NM  NM  NM  NM  NM  NM  NM  NM  NM  17-21 g  20±2 g  NM  NM  NM  20-25 g  NM  NM  NM  NM  NM  NM  NM  NM  NM  NM  25 g  NM  12-14 g  NM  NM  NM  NM  NM  NM  NM  NM  NM  NM  NM  NM  NM  NM  NM  25 g  22.8 ± 0.5 g  NM  NM  NM  NM  NM  NM  22-25 g  16-18 g  20 g  NM  NM  NM  NM  NM  NM  NM  NM  NM  NM  NM  15-22 g  25 g  NM  NM  NM  NM  NM  NM  NM  NM  NM  NM  NM  NM  NM  NM  NM  NM  NM  NM  15.2-17.3 g  NM  20-25 g  18-20 g  NM  NM  NM  NM  NM  NM  NM  NM  18 g  NM  20-22 g  NM  NM  NM  NM  NM  NM  NM  NM | NM  light/temperature controlled facility  NM  under barrier conditions  NM  under pathogen-free conditions  light/temperature controlled facility  under pathogen-free conditions  animal facility  NM  under pathogen-free conditions  NM  SPF animal facility  animal facility  NM  NM  NM  NM  under pathogen-free conditions  under pathogen-free conditions  light/temperature controlled SPF facility  NM  NM  under pathogen-free conditions  NM  NM  NM  light/temperature controlled facility  NM  NM  NM  animal facility  NM  NM  NM  NM  NM  animal facility  NM  animal facility  under pathogen-free conditions  NM  NM  under pathogen-free conditions  NM  NM  NM  NM  NM  light/temperature controlled facility  under pathogen-free conditions  light/temperature controlled facility  light/temperature controlled facility  NM  NM  light/temperature controlled facility  micro isolator cages under pathogen–free condition  light/temperature controlled SPF facility  NM  animal facility under Bsl-2 conditions  NM  SPF condition  NM  NM  NM  light/temperature controlled facility  NM  micro-isolator cages, light/temperature controlled facility  NM  NM  NM  light/temperature controlled facility  under standard specific pathogen-free conditions  NM  under pathogen-free conditions  NM  light/temperature controlled facility  NM  NM  NM  under standard specific-pathogen-free conditions  NM  NM  NM  NM  NM  light/temperature controlled facility  light/temperature controlled facility  NM  NM  light/temperature controlled facility  NM  light/temperature controlled SPF facility  NM  light/temperature controlled facility  NM  NM  light/temperature controlled SPF facility  NM  NM  NM  NM  NM  NM  NM  NM  NM  under standard specific-pathogen-free conditions | | Yes  Yes  NM  NM  NM  Yes  Yes  Yes  NM  Yes  Yes  Yes  Yes  NM  Yes  Yes  NM  Yes  Yes  NM  Yes  Yes  Yes  NM  Yes  Yes  Yes  NM  NM  Yes  Yes  Yes  Yes  Yes  NM  NM  NM  NM  Yes  NM  NM  Yes  NM  Yes  Yes  Yes  Yes  NM  Yes  Yes  Yes  Yes  Yes  Yes  Yes  Yes  Yes  Yes  NM  NM  Yes  NM  Yes  NM  Yes  Yes  Yes  Yes  Yes  NM  Yes  Yes  NM  NM  NM  Yes  Yes  Yes  Yes  NM  NM  NM  NM  NM  NM  Yes  Yes  Yes  Yes  NM  Yes  Yes  Yes  Yes  Yes  NM  Yes  Yes  Yes  NM  Yes  Yes  Yes  Yes  NM  Yes  Yes  Yes |

*NM= Not Mentioned
