# Supplementary material for: Animal models in preclinical metastatic breast cancer immunotherapy research: A systematic review and meta-analysis of efficacy outcomes
Source: PLoS One. 2025 May 7;20(5):e0322876. doi: 10.1371/journal.pone.0322876 (PMC12057864; doi:10.1371/journal.pone.0322876)
Supplement: S5 Table — (DOCX) [file pone.0322876.s005.docx]

**S5 Table. Main study design characteristics of the 100 studies included in Systematic review**

| Article | Cell number | Route of cell application | Starting time of study | Study duration |
| --- | --- | --- | --- | --- |
| 1  2  3  4  5  6  7  8  9  10  11  12  13  14  15  16  17  18  19  20  21  22  23  24  25  26  27  28  29  30  31  32  33  34  35  36  37  38  39  40  41  42  43  44  45  46  47  48  49  50  51  52  53  54  55  56  57  58  59  60  61  62  63  64  65  66  67  68  69  70  71  72  73  74  75  76  77  78  79  80  81  82  83  84  85  86  87  88  89  90  91  92  93  94  95  96  97  98  99  100  101  102  103  104  105  106  107  108 | 3×10^6^ & 10^5^  Transgenic &10^4^  for both 10^5^  10^5^  10^7^ & 5×10^6^  2-5×10^6^  2×10^7^  5×10^6^  2×10^6^  10^7^  5×10^6^  *NM  Transgenic  5×10^6^  3×10^6^  1-2×10^6^  2×10^6^  5×10^6^  5×10^6^ & 5×10^5^  NM  1.2 ×10^4^  10^7^  5 ×10^6^  10^6^  10^6^  10^6^  2-5×10^6^  10^7^  5×10^6^  10^7^  0.75 × 10^6^  10^6^  3×10^6^  3×10^6^  5×10^5^  5×10^6^  10^6^  10^6^  NM  2×10^5^  2×10^5^  2.5-5×10^5^  10^6^  NM  2.5×10^7^  5×10^6^  5×10^6^  10^7^  5×10^6^  4×10^4^  5×10^5^  5×10^6^  2 × 10^6^  NM  10^6^  10^3^  4×10^6^  2 × 10^6^  10^5^  10^4^  5×10^6^  5×10^6^  10^6^  10^6^  4×10^6^  NM  2×10^6^  2-5×10^6^  5×10^6^  4×10^6^  4-8×10^5^  5×10^6^  NM  10^6^  2-4×10^6^  10^7^  5×10^6^  3×10^6^  2×10^6^  5×10^6^  10^5^  for both 5×10^6^  4×10^6^  Transgenic  10^6^  10^6^  2-2.5×10^5^  10^6^  5×10^6^  10^6^  NM  10^6^  5×10^4^  3×10^6^  4×10^5^  Transgenic & 2×10^6^  2-5×10^5^  4×10^6^  10^5^  2×10^6^  1.5-2×10^6^  1×10^5^  3×10^7^ & 3×10^4^  5 × 10^6^  1 × 10^6^  1 × 10^5^  2× 10^6^ & 5× 10^6^  2× 10^6^ | S.C. & Mammary fat pad  Transgenic & Mammary fat pad  Intracardiac  Mammary fat pad  Mammary fat pad  S.C.  S.C.  Mammary fat pad  S.C.  S.C.  S.C.  Mammary fat pad  Transgenic  Mammary fat pad  S.C.  Mammary fat pad  Mammary fat pad  S.C.  S.C. & IV& Mammary fat pad  Mammary fat pad  Transgenic & Mammary fat pad  S.C.  S.C. & IV  S.C. & IV  S.C.  IV  Mammary fat pad  Mammary fat pad  IV  S.C.  Mammary fat pad  S.C.  S.C.  S.C.  Mammary fat pad  S.C.  S.C.  Mammary fat pad & Intracardiac  Mammary fat pad & IV  S.C.  IV& Mammary fat pad  Mammary fat pad & S.C. & Intracardiac  Mammary fat pad  Intracardiac  Mammary fat pad  S.C.  S.C.  Mammary fat pad  S.C.  Mammary fat pad  Mammary fat pad  S.C.  S.C.  S.C.  Mammary fat pad  Mammary fat pad  Mammary fat pad  Mammary fat pad  Mammary fat pad  Mammary fat pad  S.C.  S.C.  Mammary fat pad  Mammary fat pad  S.C.  NM  S.C.  S.C.  S.C.  Mammary fat pad  Mammary fat pad & IV  S.C.  Intracardiac& Transgenic  Mammary fat pad  Mammary fat pad & IV  S.C.  S.C.  Mammary fat pad & S.C.  Mammary fat pad  Mammary fat pad  Mammary fat pad  S.C.  S.C.  Transgenic  S.C.  Mammary fat pad  IV & Mammary fat pad  S.C. & IV  S.C.  Mammary fat pad  Mammary fat pad  S.C.  IV  Mammary fat pad & S.C.  S.C.  Mammary fat pad  Mammary fat pad & Intratibial  S.C.  Intracardiac  S.C.  S.C. & IV  Mammary fat pad  IV & S.C.  S.C.  S.C.  Mammary fat pad  S.C.  IV | first day  day 1/ day 10  day 1/ 14 days after inoculation  day zero/ day 15  10 & 15 days after inoculation  2 weeks after inoculation  first day  14 days after inoculation  one day after inoculation  When tumor was 20-60 mm^3^  When tumor was 100-150mm^3^  15 days after implantation  day 1 & day 8  one day after implantation  day zero & day 8 after implantation  day 8 & day 10 after implantation  when tumor was 100 mm^3^  when tumors was 50–100 mm^3^  When the tumor was 200 mm^3^  6 days after inoculation  when tumor was 100 mm^3^  when tumor was 180-250 mm^3^  one day after inoculation  NM  7 days after implantation  6 hours before inoculation  14 days after inoculation  7 days after inoculation  one day before, one day after inoculation  When tumor was 4 to 5 mm in diameter  14 days after inoculation  when tumors was 100 mm^3^  when tumor was in appropriate size  when tumor was in appropriate size  when tumor was palpable  When tumors was 200 mm^3^  day 7  when tumor was 200 mm^3^/on day 2  40 & 2 days after inoculation  7 days after inoculation  2 hour before/ 1& 6 days after inoculation  7 & 21 days after/3 days before inoculation  14 days after inoculation  7 days after inoculation  when tumors was 100 mm^3^  17 & 23 days after inoculation  7 days after implantation  10 days after inoculation  When tumor was 150 mm^3^  4 days after inoculation  7 days after inoculation  14 days after inoculation  when tumor was 70 mm^3^  when tumor was 150-250 mm^3^  when tumor was 0.4-0.55 cm in length  15-18 days after inoculation  day 1  20 & 10 days after inoculation  NM  4 days before & 3 days after inoculation  11 & 12 days after inoculation  when tumor was 100 mm^3^  14 days after inoculation  when tumor was 100 mm^3^  when tumor was 100~150 mm^3^  when tumor was 100-250 mm^3^  when tumor was 100 mm^3^  when tumor was 120 mm^3^ /200 mm^3^/400mm3  7 days after implantation  6 & 7 days after implantation  When tumor was palpable  when tumor was 150 mm^3^  when tumor was 100 & 400 mm^3^  when tumor was 100 mm^3^  when tumor was 400mm^3^  when tumors was 100 mm^3^  when tumor was 150 mm^3^  one week after inoculation  when tumor was 150 mm^3^  7 days after inoculation  7 days after inoculation  one day after the inoculation  11 days after inoculation  from 9 weeks of age  7 days after inoculation  when tumor was 100 mm^3^  when tumor was 100 mm^3^  when tumor was 80–120 mm^3^  first day  9 days after inoculation  7 days after inoculation  NM  NM  when tumor was 100 mm^3^  day 5  when tumor was visible  when tumor was 50-100 mm^3^  when tumor was 100 mm^3^  One day after inoculation  first day  when tumor was 100-200 mm^3^  When tumor was nearly 100 mm^3^  5 days & 7 days after inoculation  NM  when tumor was 100 mm^3^  When tumor was palpable  when tumor was 50 or 100 mm^3^ depending on model  first day | 21 & 32 days  21 & 28 days  28 days  14 & 21 days  35 & 48 days  23 & 35 days  21 days  41 days  21 days  41& 47 days  21 days  33 days  27 days  35 days  21 & 28 days  40 to 46 days  33 & 35 days  19 & 21 days  28 & 21 & 42 days  41 & 34 days  70 & 28 days  45 days  42 & 30 & 15 days  63 & 42 days  28 days  21 days  22 days  23 days  15 & 13 days  321 days  31 days  21 days  27 days  25 days  50 days  28 days  28 days  92 days  60 & 15 days  20 & 30 days  42 & 28 days  21 & 27 & 35 days  24 days  28 days  26 days  after 21 dose  22 & 24 days  26 days  21 days  42 days  65 days  22 days  16 days  21 days  49 days  39 days  17 days  75 & 34 days  28 days  16 & 19 days  27 & 33 days  21 days  25 days  21 days  18 days  13 & 33 days  30 days  48 & 32 & 39 days  21 days  29 days  19 & 28 & 21 & 49 days  67 & 21 days  14 & 16 & 28 days  58 days  18 & 42 & 14 days  21 days  22 & 62 & 58 days  22 days  25 & 42 days  32 days  27 days  29 & 55 days  22 days  until 15 weeks of age  21 days  30 days  18 & 23 & 56 days  8 & 30 days  21 & 24 days  23 days  28 days  21 days  14 days  42 & 28 days  37 days  28 & 25 days  35 & 70 days  35 days  35 days  14 days  21 & 25 days  23 days  30 & 34-42 days  22 days  14 days  29 days  42 & 46 & 48 days  56 days |

IV= Intravenous, S.C.= Subcutaneous, and NM= not mentioned. The tumor volume in the studies considered an approximate measure.
