# Supplementary material for: Animal models in preclinical metastatic breast cancer immunotherapy research: A systematic review and meta-analysis of efficacy outcomes
Source: PLoS One. 2025 May 7;20(5):e0322876. doi: 10.1371/journal.pone.0322876 (PMC12057864; doi:10.1371/journal.pone.0322876)
Supplement: S6 Table — (DOCX) [file pone.0322876.s006.docx]

**S6 Table. Main experiment design characteristics of the 100 studies included in Systematic review**

| Article | Therapeutic agent | Dose | Route of application |
| --- | --- | --- | --- |
| 1  2  3  4  5  6  7  8  9  10  11  12  13  14  15  16  17  18  19  20  21  22  23  24  25  26  27  28  29  30  31  32  33  34  35  36  37  38  39  40  41  42  43  44  45  46  47  48  49  50  51  52  53  54  55  56  57  58  59  60  61  62  63  64  65  66  67  68  69  70  71  72  73  74  75  76  77  78  79  80  81  82  83  84  85  86  87  88  89  90  91  92  93  94  95  96  97  98  99  100  101  102  103  104  105  106  107  108 | a small molecule as a selective survivin suppressant Named YM155  a Novel small molecule as ALK5 kinase inhibitor Named EW-7195  an Anti-Transforming Growth Factor ß anticody Named 1D11  an agent that targets LPA1 Named Debio 0719  a small molecule active-site TORC1/2 kinase inhibitor Named MLN0128  an anti-Cyr61 monoclonal antibody  a Novel hydroxamic acid-based HDAC inhibitor Named MHY218  a histone deacetylase inhibitor Named panobinostat (LBH589)  a small molecule as Anti-FAK inhibitor Named M13  a HER2-lytic hybrid peptide that target HER2  a Novel histone deacetylase inhibitor Named NK-HDAC-1  a small molecule for the uPAR·uPA interaction inhibition  granulocyte–macrophage colony-stimulating factor (GM-CSF) group  a Novel orally active non-cytotoxic inhibitor for EP4 Named RQ-00015986  a selective inhibitor of 20-HETE synthesis Named HET0016  a Novel SET antagonist Named OP449  a Novel collagen IV derived biomimetic peptide Named SP2043  a fluorescent PI3K inhibitor Named HS-133  a synthetic small molecule inhibitor of (Hsp90) Named Ganetespib  a pan-DAC inhibitor panobinostat Named LBH589  a Novel ALK-5 Kinase Inhibitor Named EW-7197  a Fab-like bispecific antibody Named HER2bsFab  a small molecule as MDM2 inhibitor Named SP-141  a Novel agent Named YL-109  a small molecule targeting the tyrosine kinase of FGFRs Named AZD4547  a MEK Inhibitor Named Selumetinib  a Novel multi-kinase inhibitor Named EC-70124  a fused TAT–DV1–BH3 polypeptide as an antagonist of CXCR4  a Novel Cyclic Peptide as a CXCR4 Antagonist Named LY2510924  a small molecule as a QSHP Inhibitor Named VR23  a small molecule as a (SRC-3/AIB1) inhibitor Named 3-phospho-bufalin  a prokaryotically and eukaryotically expressed/purified rhIL-24 protein  a Novel FGFR2 inhibitor Named Formononetin  a Novel inhibitor of VEGFR2 signaling Named Rhamnazin  a Novel covalent mTOR Inhibitor nemaed DHM25  a small molecule as a new inhibitor of angiogenesis  a small molecule as an anti-anginal drug  a neuropilin-1 transmembrane domain interfering peptide Named MTP-NRP1  a first-in-class covalent inhibitor of FGFR Named FIN-4  an immunotoxin protein targeting fibroblast activation Named aFAP-PE38  a selective inhibitor of checkpoint kinases 1 and 2 Named DN10764  a LIM kinas inhibitor Named Pyr1  a small molecule as NFAT1-MDM2 pathway inhibitor Named inulanalide  a selective small-molecule antagonist of CX3CR Named JMS-17-2  a Novel VEGF receptor 2 inhibitor Named YLL545  an anti-DLL4 monoclonal antibody Named MMGZ01  a Poly (ADP-ribose) polymerases inhibitor, Named Zj6413  a phosphatidylserine-targeting antibody Named mch1N11 (10 mpk)  a 2,4-difluoro-linker as (PARP1) inhibitor Named compound 56 and 57  a ɷ-3 17,18-epoxyeicosatetraeNMic acid analouge Named ɷ-3 epoxide C20E  a Dual Rac/Cdc42 Inhibitor Named MBQ-167  a Novel VEGF receptor–specific inhibitor Named F16  a micellar mertansine prodrug Named (cRGD-MMP)  an irreversible EGFR/HER2 dual tyrosine kinase inhibitor Named SHR1258  a Novel Pyrrolopyrimidines as Mps1/TTK Kinase Inhibitor  a parasite-derived peptide adjuvant of 18 amino acid-length Named GK-1  a monoclonal anti–IL-8 antibody Named HuMax-IL8  a Usnic Acid Benzylidene Analogues as Rapamycin Inhibitors Named 52  an antisense oligonucleotide targeting TGF-b2 Named ISTH0047 & ISTH-C3  an attenuated Listeria monocytogenes infected MDSC Named Listeria-Mage-b  a Dual TTK Protein Kinase/CLK2 Inhibitor Named CC-671  an orally inhibitor of Signal Transducer and activator of Transcription 3  a new SK1 inhibitor Named SK-F  a small molecule activator of NM23/NDPK Named NMac1  a Novel PLK4 inhibitor Named YLT-11  a Novel multi‑kinase inhibitor Named T03  a Novel potent VEGF receptor 2 Kinase Inhibitor Named Isomangiferin  a potent and selective TTK and CLK1/2 Inhibitor Named CC-67  a small molecule inhibitor of the STAT3 signaling pathway Named Bt354  a 212Pb-Labeled antibody Named pb-F3-C25 and pb-225-C28  a Choline Kinase Alpha Inhibitor Named EB-3D  a Phosphorylated-p68 Inhibitor Named RX-5902  a small molecule that inhibits TGFβ/BMP signaling Named ZL170  a Novel Humanized Monoclonal antibody Named hSFRP2 mAb  a MEK1 inhibitor Named E6201  a BET inhibitor Named JQ1  a Novel small molecule as tyrosine kinase inhibitor Named DCC-2036  a Novel IL-6/GP130 inhibitor Named Bazedoxifene  a small molecule inhibitor for LIFR signaling Named EC359  a small molecule inhibitor Named WX2–43  a C-terminal HSP90 inhibitor Named L80  a TIP60 Inhibitor Named TH1834  a MTHFD2 Inhibitor Named DS18561882 compound 18 and 2  a monoclonal antibody Named mAb3F  a Novel specific Anti-CD73 antibody Named (3F7)  a Jab1 inhibitor Named CSN5i-3  a Tubulin inhibitor Named VERU-111  a Nanometer-sized inhibitor of a pro-cancer protease Named CQD-KD1  a Novel triple-acting PIM/PI3K/mTOR inhibitor Named IBL-302  a Novel Non-peptide small molecule as STAT3 inhibitor Named LLY17  a regulator of inflammatory cytokines Named BML-111  a MyD88 inhibitor Named TJ-M2010-2  a small molecule for targeting CXCR4/CXCL12 axis Named compound IIIe  a Novel hydroxamic acid-based HDAC inhibitor Named Pracinastat (SB939)  a cell penetrating and interfering peptide targeting PP2A/SET  a neutralizing antibody to Robo1 Named R5  a uPAR antibody Named huATN-658  a Novel small molecule STAT3 inhibitor Named SLSI-1216 and SLSI-1  a small molecule inhibitor Named LMW  a small molecule Fam20 inhibitor Named 3r  a highly selective VEGFR3 Inhibitor named compound 38k  an anti- TGF-β/PD-L1bispecificantibody named BiTP  a TNBC stemness inhibitor named YH677  a specific RAGE-binding peptide named RP7  a novel CDK9-Cyclin T1 pr-pr inhibitor named compound B19  a 9S1R nullomer peptide  a novel Fc- engineered cathepsin D antibody named F1M1-Fc ^+^  a potent PAK2 inhibitor named FRAX486 | 5 & 2 mg/kg  40 mg/kg  10 mg/kg  25 & 50 mg/kg  0.3 & 3 mg/kg  5 mg/kg  10 mg/kg  10 mg/kg  30 & 50 mg/kg  3 mg/kg  100, 50, 30, 10 & 3 mg/kg  200 mg/kg  200 µl  100 mg/kg  10 mg/kg  5 mg/kg  10 & 20 mg/kg  5 mg/kg & 200µl  100 & 150 mg/kg  10 mg/kg  40, 5 & 20 mg/kg  5 mg/kg  40 & 20 mg/kg  15 mg/kg  5 mg/kg  50 mg/kg  18 mg/kg  1.2 mM/100μL  3 mg/kg  30 mg/kg  0.75 mg/kg  2μg  100 mg/kg  200 mg/kg  NM  25 mg/kg  0.400 mg/kg  1 μg/kg & 1.5 μg/kg  25 mg/kg  0.5 mg/kg  10 & 20 mg/kg  10 mg/Kg  30 mg/kg  10 mg/kg  50 mg/kg  5 & 10 mg/kg  25 & 50 mg/kg  100μl  1, 10 & 30 mg/kg  0.05 & 0.5 mg/kg  1 & 10 mg/kg  100 mg/kg  0.8 & 1.6 mg equiv./kg  5, 10 & 20 mg/kg  10 mg/kg  10, 50 & 100µg  200 µg  10 mg/kg  20 mg/kg  10^4^ & 0.5 ×10^7^ CFU  10 & 20 mg/kg  5 mg/kg  5 mg/kg  10 mg/kg  30 & 90 mg/kg  50 & 100 mg/kg  10 mg/kg  5, 10, 20 & 40 mg/kg  10, 20 & 40 mg/kg  14, 0.30, 0.48 & 0.33 MBq  1 & 2.5 mg/kg  160, 320 & 600 mg/kg  80, 40 & 20 mg/kg  4 mg/kg  30 mg/kg  50 mg/kg  100 & 150 mg/kg  8.8 & 4.4 mg/kg  5 & 10 mg/kg  40 & 80 mg/kg  20 mg/kg  50 mg/kg  30, 100 & 300 mg/kg  50 µg per mouse  10 mg/kg  100 mg/kg  5, 10 & 12.5 mg/kg  0.64 & 6.4 mg kg -1  50 mg/kg  5 mg/kg  1 mg/kg (0.1ml)  NM  20 mg/kg  25 mg/kg  5 mg/kg  1 mg/mouse  10 mg·kg−1  5 and 10 mg/kg  5 mg/kg  25 & 50 mg/kg  25 & 50 mg/kg  9 mg/kg  1 & 10 mg/kg  15 & 30 mg/kg  12.5 & 25 mg/kg  100 mg/kg  15 mg/kg  20 mg/kg | OP  IP  NM  PO  IG  IP  IP  IP  IP  IV  IP  IG  S.C. & IV  IV  IG  IP  IP  IT & IG  IV  IP  IP & PO  IP  IP  S.C.  IP  IG  IV  IT  S.C.  IP  S.C.  IT  IG  IG  IP  IP  IG  IP  PO  IV  IP  IP  IP  IP  PO  IV  IG  IP  PO  IP  IP  IP  IV  PO  IP  IV  IP  IP  S.C.  IP  IV  PO  IP  PO  IG  PO  IP  IV  IG  IV  IP  PO  IP  IV  IV  IP  IG  PO  S.C.  IP  IP  S.C.  PO  IP  IP  PO  PO  S.C. & IV  PO  PO  NM  NM  IP  PO  IP  IP  IP  IP  IP  IG  PO  IP  IV  IV  IP  IP  IP  IG |

IP= Intraperitoneal, IG= Intragastric, PO= Orally, OP= Osmotic Pump, IT= Intratumoral, S.C.= Subcutaneous, IV= Intravenous, and NM= not mentioned.
