# Supplementary material for: Animal models in preclinical metastatic breast cancer immunotherapy research: A systematic review and meta-analysis of efficacy outcomes
Source: PLoS One. 2025 May 7;20(5):e0322876. doi: 10.1371/journal.pone.0322876 (PMC12057864; doi:10.1371/journal.pone.0322876)
Supplement: S7 Table — (DOCX) [file pone.0322876.s007.docx]

**S7 Table. Reporting quality assessment using the ARRIVE guidelines**

**The ARRIVE questions:**

A) *Study design:* Are all experimental and control groups clearly identified? Is the experimental unit clearly identified?

B) *Sample size:* Is the exact number of experimental units in each group at the start of the study provided? Is the method by which the sample size was chosen explained?

C) *Inclusion & exclusion criteria:* Are the criteria used for including and excluding animals, experimental units, or data points provided? Are any exclusions of animals, experimental units, or data points reported, or is there a statement indicating that there were no exclusions?

D) *Randomization:* Is the method by which experimental units were allocated to control and treatment groups described?

E) *Blinding:* Is it clear whether researchers were aware of, or blinded to, the group allocation at any stage of the experiment or data analysis?

F) *Outcome measures:* For all experimental outcomes presented, are details provided of exactly what parameter was measured?

G) *Statistical methods:* Is the statistical approach used to analyses each outcome detailed? Is there a description of any methods used to assess whether data met statistical assumptions?

H) *Experimental animals:* Are all species of animal used specified? Is the sex of the animals specified? Is at least one of age, weight or developmental stage of the animals specified?
I) *Experimental procedures:* Are both the timing and frequency with which procedures took place specified? Are details of acclimatization periods to experimental locations provided?

J) *Results:* Are descriptive statistics for each experimental group provided, with a measure of variability? Is the effect size and confidence interval provided?

| Reference | Year | A | B | C | D | E | F | G | H | I | J |
| --- | --- | --- | --- | --- | --- | --- | --- | --- | --- | --- | --- |
| 1  2  3  4  5  6  7  8  9  10  11  12  13  14  15  16  17  18  19  20  21  22  23  24  25  26  27  28  29  30  31  32  33  34  35  36  37  38  39  40  41  42  43  44  45  46  47  48  49  50  51  52  53  54  55  56  57  58  59  60  61  62  63  64  65  66  67  68  69  70  71  72  73  74  75  76  77  78  79  80  81  82  83  84  85  86  87  88  89  90  91  92  93  94  95  96  97  98  99  100  101  102  103  104  105  106  107  108 | 2011  2011  2011  2012  2012  2012  2012  2012  2013  2013  2013  2013  2013  2013  2014  2014  2014  2014  2014  2014  2014  2014  2014  2014  2014  2015  2015  2015  2015  2015  2015  2015  2015  2015  2015  2015  2015  2016  2016  2016  2016  2016  2016  2016  2016  2016  2016  2016  2017  2017  2017  2017  2017  2017  2017  2017  2017  2017  2017  2017  2017  2017  2018  2018  2018  2018  2018  2018  2018  2018  2018  2019  2019  2019  2019  2019  2019  2019  2019  2019  2019  2019  2019  2019  2019  2019  2020  2020  2020  2020  2020  2020  2020  2020  2020  2020  2020  2020  2021  2021  2021  2022  2023  2023  2023  2024  2024  2024 | FR  FR  FR  FR  FR  FR  FR  FR  FR  FR  FR  FR  FR  FR  FR  FR  FR  FR  FR  FR  FR  FR  FR  FR  FR  FR  FR  FR  FR  FR  FR  FR  FR  FR  FR  FR  FR  FR  FR  FR  FR  FR  FR  FR  FR  FR  FR  FR  FR  FR  FR  FR  FR  FR  FR  FR  FR  FR  FR  FR  FR  FR  FR  FR  FR  FR  FR  FR  FR  FR  FR  FR  FR  FR  FR  FR  FR  FR  FR  FR  FR  FR  FR  FR  FR  FR  FR  FR  FR  FR  FR  FR  FR  FR  FR  FR  FR  FR  FR  FR  FR  FR  FR  FR  FR  FR  FR  FR | PR  PR  PR  NR  PR  PR  PR  PR  PR  PR  PR  PR  PR  PR  PR  PR  PR  PR  PR  PR  PR  PR  PR  PR  PR  PR  PR  PR  PR  PR  PR  PR  PR  PR  PR  PR  PR  PR  PR  PR  PR  PR  PR  PR  PR  PR  PR  PR  PR  PR  PR  NR  PR  PR  PR  PR  PR  PR  PR  PR  PR  PR  PR  PR  PR  PR  PR  PR  PR  PR  PR  PR  PR  PR  PR  PR  PR  PR  PR  PR  PR  PR  PR  PR  PR  PR  PR  PR  PR  PR  PR  PR  PR  NR  PR  PR  PR  PR  PR  PR  PR  PR  PR  PR  PR  PR  PR  PR | PR  PR  PR  PR  PR  PR  PR  PR  PR  PR  PR  PR  PR  PR  PR  PR  PR  PR  PR  PR  PR  PR  PR  PR  PR  PR  PR  PR  PR  PR  PR  PR  PR  PR  PR  PR  PR  PR  PR  PR  PR  PR  PR  PR  PR  PR  PR  PR  PR  PR  PR  PR  PR  PR  PR  PR  PR  PR  PR  PR  PR  PR  PR  PR  PR  PR  PR  PR  PR  PR  PR  PR  PR  PR  PR  PR  PR  PR  PR  PR  PR  PR  PR  PR  PR  PR  PR  PR  PR  PR  PR  PR  PR  PR  PR  PR  PR  PR  PR  PR  PR  PR  PR  PR  PR  PR  PR  PR | PR  PR  NR  NR  NR  PR  PR  PR  PR  PR  PR  PR  PR  NR  NR  PR  NR  PR  PR  NR  PR  PR  PR  NR  PR  PR  PR  NR  NR  PR  PR  PR  PR  PR  NR  NR  NR  NR  PR  NR  NR  PR  NR  PR  PR  PR  PR  NR  PR  PR  PR  PR  PR  PR  PR  PR  PR  PR  NR  NR  PR  NR  PR  NR  PR  PR  PR  NR  PR  NR  PR  PR  NR  NR  NR  PR  PR  PR  PR  NR  NR  NR  NR  NR  NR  PR  PR  PR  PR  NR  PR  PR  PR  PR  PR  NR  PR  PR  PR  NR  PR  PR  PR  PR  NR  PR  PR  PR | NR  NR  NR  NR  NR  NR  NR  NR  NR  NR  NR  NR  NR  NR  NR  NR  NR  NR  NR  NR  NR  NR  NR  NR  NR  NR  NR  NR  NR  NR  NR  NR  NR  NR  NR  NR  NR  NR  NR  NR  NR  NR  NR  NR  NR  NR  NR  NR  NR  NR  NR  NR  NR  NR  NR  NR  NR  NR  NR  NR  NR  NR  NR  NR  NR  NR  NR  NR  NR  NR  NR  NR  NR  NR  NR  NR  NR  NR  NR  NR  NR  NR  NR  NR  NR  NR  NR  NR  NR  NR  NR  NR  NR  NR  NR  NR  NR  NR  NR  NR  NR  NR  NR  NR  NR  NR  NR  NR | FR  FR  FR  FR  FR  FR  FR  FR  FR  FR  FR  FR  FR  FR  FR  FR  FR  FR  FR  FR  FR  FR  FR  FR  FR  FR  FR  FR  FR  FR  FR  FR  FR  FR  FR  FR  FR  FR  FR  FR  FR  FR  FR  FR  FR  FR  FR  FR  FR  FR  FR  FR  FR  FR  FR  FR  FR  FR  FR  FR  FR  FR  FR  FR  FR  FR  FR  FR  FR  FR  FR  FR  FR  FR  FR  FR  FR  FR  FR  FR  FR  FR  FR  FR  FR  FR  FR  FR  FR  FR  FR  FR  FR  FR  FR  FR  FR  FR  FR  FR  FR  FR  FR  FR  FR  FR  FR  FR | FR  FR  FR  FR  FR  FR  FR  FR  FR  FR  FR  FR  FR  FR  FR  FR  FR  FR  FR  FR  FR  FR  FR  FR  FR  FR  FR  FR  FR  FR  FR  FR  FR  FR  FR  FR  FR  FR  FR  FR  FR  FR  FR  FR  FR  FR  FR  FR  FR  FR  FR  FR  FR  FR  FR  FR  FR  FR  FR  FR  FR  FR  FR  FR  FR  FR  FR  FR  FR  FR  FR  FR  FR  FR  FR  FR  FR  FR  FR  FR  FR  FR  FR  FR  FR  FR  FR  FR  FR  FR  FR  FR  FR  FR  FR  FR  FR  FR  FR  FR  FR  FR  FR  FR  FR  FR  FR  FR | PR  PR  PR  PR  PR  PR  PR  PR  PR  FR  FR  PR  PR  PR  FR  PR  PR  PR  PR  PR  PR  PR  PR  PR  PR  FR  PR  FR  PR  PR  PR  PR  PR  PR  PR  PR  PR  PR  PR  PR  PR  PR  PR  FR  PR  PR  PR  PR  PR  PR  PR  FR  FR  FR  PR  PR  PR  PR  PR  PR  PR  PR  PR  PR  PR  FR  FR  PR  PR  PR  PR  PR  PR  PR  PR  PR  PR  PR  PR  PR  PR  PR  PR  PR  PR  FR  PR  PR  FR  PR  PR  PR  PR  PR  PR  PR  PR  FR  PR  FR  PR  PR  PR  PR  PR  PR  PR  PR | FR  PR  PR  PR  PR  PR  PR  PR  PR  FR  FR  PR  PR  PR  PR  PR  PR  FR  PR  PR  PR  PR  PR  PR  FR  FR  PR  PR  PR  FR  PR  FR  PR  PR  PR  FR  PR  FR  PR  FR  PR  FR  PR  PR  FR  FR  FR  FR  FR  PR  PR  PR  FR  PR  PR  FR  PR  PR  PR  PR  PR  FR  PR  PR  FR  PR  FR  PR  FR  FR  PR  FR  PR  FR  FR  FR  FR  FR  PR  FR  FR  PR  PR  PR  PR  FR  FR  FR  PR  PR  PR  PR  FR  PR  PR  FR  PR  FR  PR  FR  FR  FR  FR  FR  FR  FR  FR  FR | FR  FR  FR  FR  FR  FR  FR  FR  FR  FR  FR  FR  FR  FR  FR  FR  FR  FR  FR  FR  FR  FR  FR  FR  FR  FR  FR  FR  FR  FR  FR  FR  FR  FR  FR  FR  FR  FR  FR  FR  FR  FR  FR  FR  FR  FR  FR  FR  FR  FR  FR  FR  FR  FR  FR  FR  FR  FR  FR  FR  FR  FR  FR  FR  FR  FR  FR  FR  FR  FR  FR  FR  FR  FR  FR  FR  FR  FR  FR  FR  FR  FR  FR  FR  FR  FR  FR  FR  FR  FR  FR  FR  FR  FR  FR  FR  FR  FR  FR  FR  FR  FR  FR  FR  FR  FR  FR  FR |

***FR= fully reported if all the information for each criterion addressed in the ARRIVE Essential 10 guideline was fully reported, **PR=partially reported if only some of the information for each criterion addressed in the ARRIVE Essential 10 guideline was reported, and ***NR= not reported if none of the information for each criterion addressed in the ARRIVE Essential 10 guideline was reported.**
