# Supplementary material for: Animal models in preclinical metastatic breast cancer immunotherapy research: A systematic review and meta-analysis of efficacy outcomes
Source: PLoS One. 2025 May 7;20(5):e0322876. doi: 10.1371/journal.pone.0322876 (PMC12057864; doi:10.1371/journal.pone.0322876)
Supplement: S8 Table — (DOCX) [file pone.0322876.s008.docx]

**S8 Table. Multivariable meta-regression analyses for primary tumor volume dataset**

Mixed-Effects Model (k = 193; tau^2^ estimator: REML)

tau^2^ = 256.3233 (SE = 37.1379), I^2^ =84.62%, R^2^ =9.10%.

Test for Residual Heterogeneity: QE(df = 190) = 1375.8701, p-value < .0001

Test of Moderators (coefficients 2:24): F(df1 = 23, df2 = 190) = 1.6541, p-value = = 0.0363

| **Variable** | **Estimate (coefficient)** | **SE** | **P value** | **95% confidence intervals** | | **t value** |
| --- | --- | --- | --- | --- | --- | --- |
| Intercept | 44.9081 | 12.5687 | 0.0004 *** | 20.1161 | 69.7002 | 3.5730 |
| Strain BALB/c nude | 13.6699 | 6.1989 | 0.0286 * | 1.4423 | 25.8974 | 2.2052 |
| Strain BALB/c | 2.8696 | 8.5480 | 0.7375 | -13.9915 | 19.7307 | 0.3357 |
| Strain C.B.17/SCID | 17.8646 | 8.4923 | 0.0367 * | 1.1132 | 34.6159 | 2.1036 |
| Strain NSG | 11.2261 | 8.2459 | 0.1750 | -5.0392 | 27.4913 | 1.3614 |
| Strain SCID | 13.8360 | 12.5598 | 0.2720 | -10.9386 | 38.6106 | 1.1016 |
| Strain transgenic | -7.8036 | 14.2043 | 0.5834 | -35.8219 | 20.2148 | -0.5494 |
| Induction method 4T1 | -4.8966 | 8.6616 | 0.5725 | -21.9818 | 12.1886 | -0.5653 |
| Induction method MDA-MB-231 | -13.7279 | 4.7925 | 0.0046 ** | -23.1811 | 4.2746 | -2.8645 |
| Induction method MC-7 | -7.5553 | 6.2446 | 0.2278 | -19.8729 | 4.7623 | -1.2099 |
| Induction method BT-474 | 7.8537 | 9.1459 | 0.3916 | 10.1868 | 25.8942 | 0.8587 |
| Induction method MDA-MB-436 | -3.8876 | 8.8874 | 0.6623 | -21.4183 | 13.6430 | -0.4374 |
| Induction method MDA-MB-468 | -7.1727 | 9.9265 | 0.4708 | -26.7530 | 12.4076 | -0.7226 |
| Induction method Cal-51 | -0.3984 | 11.6664 | 0.9728 | -23.4108 | 22.6140 | -0.0342 |
| Induction method SUM159 | 0.6162 | 8.6507 | 0.9433 | -16.4476 | 17.6800 | 0.0712 |
| Induction method PDX | 3.1841 | 8.9130 | 0.7213 | -14.3971 | 20.7652 | 0.3572 |
| Cell application route S.C. | 3.1283 | 6.5141 | 0.6316 | -9.7209 | 15.9776 | 0.4802 |
| Cell application route Mammary fat pad | -1.5269 | 6.8847 | 0.8247 | -15.1070 | 12.0533 | -0.2218 |
| Drug administration route IP | 15.4908 | 8.9218 | 0.0841 | -2.1078 | 33.0893 | 1.7363 |
| Drug administration route Oral | 5.5266 | 8.7698 | 0.5293 | -11.7720 | 22.8252 | 0.6302 |
| Drug administration route IV | 6.2065 | 9.1741 | 0.4995 | -11.8896 | 24.3027 | 0.6765 |
| Drug administration route S.C. | 14.1759 | 10.9781 | 0.1982 | -7.4787 | 35.8305 | 1.2913 |
| Drug administration route N.M. | -2.7436 | 11.9093 | 0.8180 | -26.2349 | 20.7477 | 0.2304 |
| Tumor model Syngeneic | -0.7771 | 9.2809 | 0.9334 | -19.0838 | 17.5297 | -0.0837 |

**Significant codes: 0 ‘***’ 0.001 ‘**’ 0.01 ‘*’ 0.05 ‘.’**
