# Supplementary material for: Animal models in preclinical metastatic breast cancer immunotherapy research: A systematic review and meta-analysis of efficacy outcomes
Source: PLoS One. 2025 May 7;20(5):e0322876. doi: 10.1371/journal.pone.0322876 (PMC12057864; doi:10.1371/journal.pone.0322876)
Supplement: S9 Table — (DOCX) [file pone.0322876.s009.docx]

**S9 Table. Multivariable meta-regression analyses for metastatic lung nodules number dataset**

Mixed-Effects Model (k = 51; tau^2^ estimator: REML)

tau^2^ = 1.6176 (SE = 0.6021), I^2^ =78.17%, R^2^ =18.01%.

Test for Residual Heterogeneity: QE(df = 35) = 132.7078, p-value < .0001

Test of Moderators (coefficients 2:16): F(df1 = 15, df2 = 35) = 1.3944, p-value = 0.2040

| **Variable** | **Estimate (coefficient)** | **SE** | **P value** | **95% confidence intervals** | | **t value** |
| --- | --- | --- | --- | --- | --- | --- |
| Intercept | 3.6871 | 1.8694 | 0.0565 **^.^** | -0.1080 | 7.4822 | 1.9724 |
| Strain BALB/c nude | -0.4208 | 1.0079 | 0.6789 | -2.4670 | 1.6254 | -0.4175 |
| Strain NSG | -2.2318 | 1.2138 | 0.0745 | -4.6960 | 0.2324 | -1.8386 |
| Strain other | -0.0272 | 1.5080 | 0.9857 | -3.0886 | 3.0343 | -0.0180 |
| Strain transgenic | -1.0576 | 2.1270 | 0.6221 | -5.3756 | 3.2603 | -0.4972 |
| Induction method MDA-MB-231 | -1.0342 | 2.0581 | 0.6185 | -5.2123 | 3.1439 | -0.5025 |
| Induction method other | -1.5658 | 1.1179 | 0.1701 | -3.8354 | 0.7037 | -1.4007 |
| Induction method PDX | -2.8936 | 2.4578 | 0.2470 | -7.8832 | 2.0959 | -1.1773 |
| Cell application route Left cardiac ventricle | -1.1476 | 1.3196 | 0.3904 | -3.8265 | 1.5314 | -0.8696 |
| Cell application route Mammary fat pad | -0.7007 | 0.7138 | 0.3330 | -2.1497 | 0.7484 | -0.9817 |
| Cell application route other | -0.6082 | 1.1816 | 0.6100 | -3.0069 | 1.7906 | -0.5147 |
| Drug administration route IV | 1.2413 | 1.1547 | 0.2898 | -1.1030 | 3.5855 | 1.0749 |
| Drug administration route N.M. | 0.4407 | 1.0779 | 0.6851 | -1.7476 | 2.6289 | 0.4088 |
| Drug administration route Oral | 2.5527 | 1.3745 | 0.0717 | -0.2376 | 5.3431 | 1.8573 |
| Drug administration route S.C. | -1.4423 | 1.0240 | 0.1678 | -3.5211 | 0.6366 | -1.4085 |
| Tumor model Syngeneic | -0.7082 | 1.8079 | 0.6976 | -4.3783 | 2.9620 | -0.3917 |

**Significant codes: 0 ‘***’ 0.001 ‘**’ 0.01 ‘*’ 0.05 ‘.’**
