# Supplementary material for: Animal models in preclinical metastatic breast cancer immunotherapy research: A systematic review and meta-analysis of efficacy outcomes
Source: PLoS One. 2025 May 7;20(5):e0322876. doi: 10.1371/journal.pone.0322876 (PMC12057864; doi:10.1371/journal.pone.0322876)
Supplement: S10 Fig — (DOCX) [file pone.0322876.s010.docx]

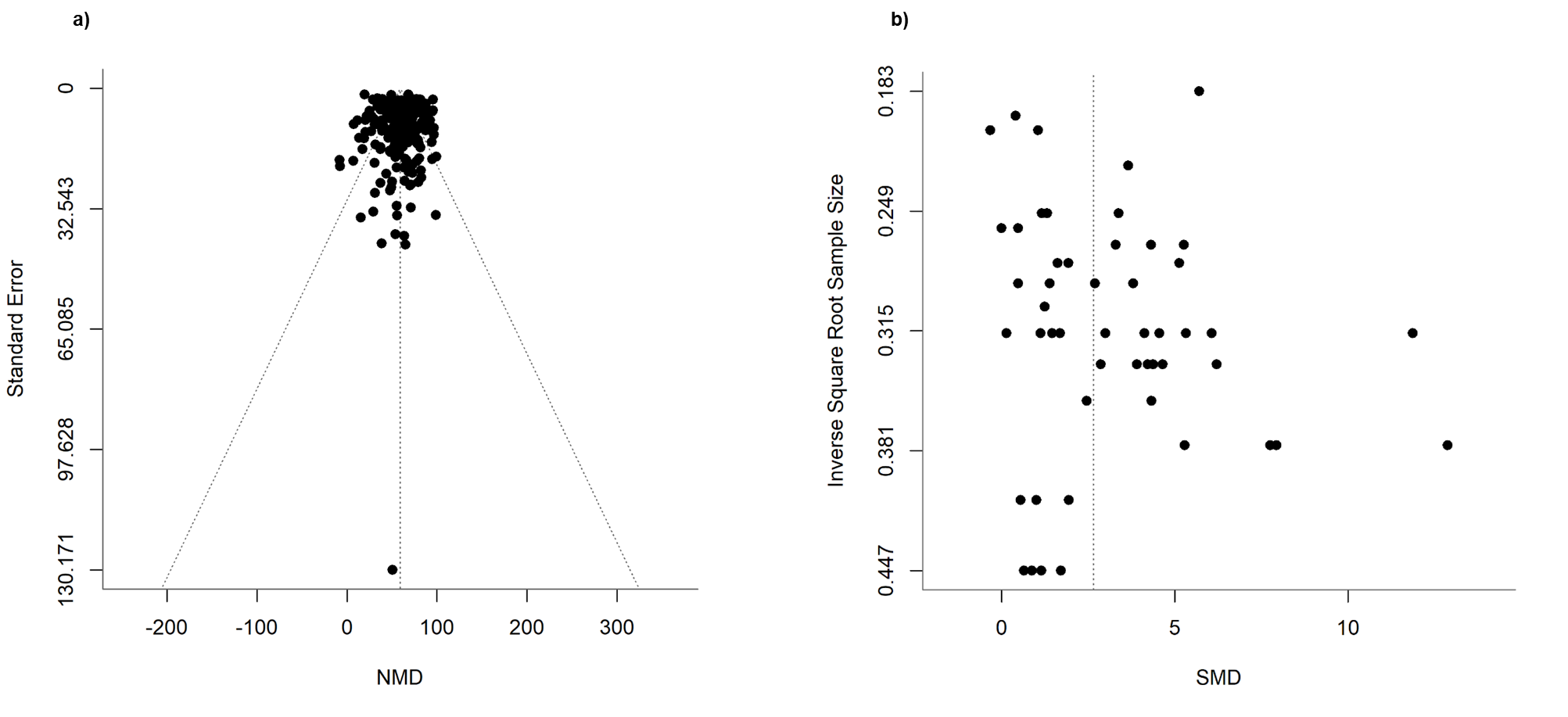


**S10 Fig. Funnel plots of immunotherapy outcomes:** a) Primary tumor volume dataset , and b) Metastasis nodule number dataset. The closed dots indicate the observed studies. The x-axis shows the observed outcome as normalized mean difference or the standardized mean difference with the y-axis representing the standard error or the inverse square root of the sample size respectively. The two dotted lines in either side of plot (a) represent the 95% confidence intervals. The short-dash vertical line indicates the effect sizes under the random effects model: a) NMD=58.73% and b) SMD=2.63.
