# Supplementary material for: Animal models in preclinical metastatic breast cancer immunotherapy research: A systematic review and meta-analysis of efficacy outcomes
Source: PLoS One. 2025 May 7;20(5):e0322876. doi: 10.1371/journal.pone.0322876 (PMC12057864; doi:10.1371/journal.pone.0322876)
Supplement: S11 File — (DOCX) [file pone.0322876.s011.docx]

**S11 File. List of the included studies in Systematic review and Meta-analyses:**

1. Yamanaka K, Nakata M, Kaneko N, Fushiki H, Kita A, Nakahara T, et al. YM155, a selective survivin suppressant, inhibits tumor spread and prolongs survival in a spontaneous metastatic model of human triple-negative breast cancer. Int J Oncol. 2011;39(3):569-75.
2. Park CY, Son JY, Jin CH, Nom JS, Kim DK, Sheen YY. EW-7195, a novel inhibitor of ALK5 kinase inhibits EMT and breast cancer metastasis to lung. Eur J Cancer. 2011;47(17):2642-53.
3. Biswas S, Nyman JS, Alvarez J, Chakrabarti A, Ayres A, Sterling J, et al. Anti-transforming growth factor ß antibody treatment rescues bone loss and prevents breast cancer metastasis to bone. PLoS One. 2011;6(11).
4. David M, Ribeiro J, Descotes F, Serre CM, Barbier M, Murone M, et al. Targeting lysophosphatidic acid receptor type 1 with Debio 0719 inhibits spontaneous metastasis dissemination of breast cancer cells independently of cell proliferation and angiogenesis. Int J Oncol. 2012;40(4):1133-41.
5. Gökmen-Polar Y, Liu Y, Toroni RA, Sanders KL, Mehta R, Badve S, et al. Investigational drug MLN0128, a novel TORC1/2 inhibitor, demonstrates potent oral antitumor activity in human breast cancer xenograft models. Breast Cancer Res Treat. 2012;136(3):673-82.
6. Lin J, Huo R, Wang L, Zhou Z, Sun Y, Shen B, et al. A novel anti-Cyr61 antibody inhibits breast cancer growth and metastasis in vivo. Cancer Immunol Immunother. 2012;61(5):677-87.
7. Park JH, Ahn MY, Kim TH, Yoon S, Kang KW, Lee J, et al. A new synthetic HDAC inhibitor, MHY218, induces apoptosis or autophagy-related cell death in tamoxifen-resistant MCF-7 breast cancer cells. Invest New Drugs. 2012;30(5):1887-98.
8. Tate CR, Rhodes LV, Segar HC, Driver JL, Pounder FN, Burow ME, et al. Targeting triple-negative breast cancer cells with the histone deacetylase inhibitor panobinostat. Breast Cancer Res. 2012;14(3).
9. Golubovskaya VM, Palma NL, Zheng M, Ho B, Magis A, Ostrov D, et al. A small-molecule inhibitor, 5'-O-tritylthymidine, targets FAK and Mdm-2 interaction, and blocks breast and colon tumorigenesis in vivo. Anticancer Agents Med Chem. 2013;13(4):532-45.
10. Kawamoto M, Horibe T, Kohno M, Kawakami K. HER2-targeted hybrid peptide that blocks HER2 tyrosine kinase disintegrates cancer cell membrane and inhibits tumor growth in vivo. Mol Cancer Ther. 2013;12(4):384-93.
11. Li ZH, Zhang XB, Han XQ, Feng CR, Wang FS, Wang PG, et al. Antitumor effects of a novel histone deacetylase inhibitor NK-HDAC-1 on breast cancer. Oncol Rep. 2013;30(1):499-505.
12. Mani T, Wang F, Knobe WE, Sinn AL, Khan M, Jo I, et al. Small-molecule inhibition of the uPAR·uPA interaction: synthesis, biochemical, cellular, in vivo pharmacokinetics and efficacy studies in breast cancer metastasis. Bioorg Med Chem. 2013;21(7):2145-55.
13. Liu X, Hu J, Cao W, Qu H, Wang Y, Ma Z, et al. Effects of two different immunotherapies on triple-negative breast cancer in animal model. Cell Immunol. 2013;284(1):111-8.
14. Ma X, Holt D, Kundu N, Reader J, Goloubeva O, Take Y, et al. A prostaglandin E (PGE) receptor EP4 antagonist protects natural killer cells from PGE2-mediated immunosuppression and inhibits breast cancer metastasis. Oncoimmunology. 2013;2(1).
15. Borin TF, Zuccari DA, Jardim-Perassi BV, Ferreira LC, Iskander AS, Varma NR, et al. HET0016, a selective inhibitor of 20-HETE synthesis, decreases pro-angiogenic factors and inhibits growth of triple-negative breast cancer in mice. PLoS One. 2014;9(12).
16. Janghorban M, Farrell AS, Allen-Petersen BL, Pelz C, Daniel CJ, Oddo J, et al. Targeting c-MYC by antagonizing PP2A inhibitors in breast cancer. Proc Natl Acad Sci U S A. 2014;111(25):9157-62.
17. Lee E, Lee SJ, Koskimaki JE, Han Z, Pandey NB, Popel AS. Inhibition of breast cancer growth and metastasis by a biomimetic peptide. Sci Rep. 2014;4:7139.
18. Lee JH, Jung KH, Lee H, Son MK, Yun SM, Ahn SH, et al. HS-133, a novel fluorescent phosphatidylinositol 3-kinase inhibitor as a potential imaging and anticancer agent for targeted therapy. Oncotarget. 2014;5(20):10180-97.
19. Proia DA, Zhang C, Sequeira M, Jimenez JP, He S, Spector N, et al. Preclinical activity profile and therapeutic efficacy of the HSP90 inhibitor ganetespib in triple-negative breast cancer. Clin Cancer Res. 2014;20(2):413-24.
20. Rhodes LV, Tate CR, Segar HC, Burks HE, Phamduy TB, Hoang V, et al. Suppression of triple-negative breast cancer metastasis by pan-DAC inhibitor panobinostat via inhibition of ZEB family of EMT master regulators. Breast Cancer Res Treat. 2014;145(3):593-604.
21. Son JY, Park SY, Kim SJ, Lee SJ, Park SA, Kim MJ, et al. EW-7197, a novel ALK-5 kinase inhibitor, potently inhibits breast to lung metastasis. Mol Cancer Ther. 2014;13(7):1704-16.
22. Turini M, Chames P, Bruhns P, Baty D, Kerfelec B. An FcγRIII-engaging bispecific antibody expands the range of HER2-expressing breast tumors eligible to antibody therapy. Oncotarget. 2014;5(14):5304-19.
23. Wang W, Qin JJ, Voruganti S, Srivenugopal KS, Nog S, Patil S, et al. The pyrido[b]indole MDM2 inhibitor SP-141 exerts potent therapeutic effects in breast cancer models. Nat Commun. 2014;5:5086.
24. Hiyoshi H, Goto N, Tsuchiya M, Iida K, Nakajima Y, Hirata N, et al. 2-(4-Hydroxy-3-methoxyphenyl)-benzothiazole suppresses tumor progression and metastatic potential of breast cancer cells by inducing ubiquitin ligase CHIP. Sci Rep. 2014;4:6692.
25. Liu L, Ye TH, Han YP, Song H, Zhang YK, Xia Y, et al. Reductions in myeloid-derived suppressor cells and lung metastases using AZD4547 treatment of a metastatic murine breast tumor model. Cell Physiol Biochem. 2014;33(3):633-45.
26. Bartholomeusz C, Xie X, Pitner MK, Kondo K, Dadbin A, Lee J, et al. MEK inhibitor selumetinib (AZD6244; ARRY-142886) prevents lung metastasis in a triple-negative breast cancer xenograft model. Mol Cancer Ther. 2015;14(12):2773-81.
27. Cuenca-López MD, Serrano-Heras G, Montero JC, Corrales-Sánchez V, Gomez-Juarez M, Gascón-Escribano MJ, et al. Antitumor activity of the novel multi-kinase inhibitor EC-70124 in triple-negative breast cancer. Oncotarget. 2015;6(29):27923-37.
28. Liang AL, Qian HL, Zhang TT, Zhou N, Wang HJ, Men XT, et al. Bifunctional fused polypeptide inhibits the growth and metastasis of breast cancer. Drug Des Devel Ther. 2015;9:5671-86.
29. Peng SB, Zhang XY, Paul D, Kays LM, Gough W, Stewart J, et al. Identification of LY2510924, a novel cyclic peptide CXCR4 antagonist that exhibits antitumor activities in solid tumor and breast cancer metastatic models. Mol Cancer Ther. 2015;14(2):480-90.
30. Pundir S, Vu HY, Solomon VR, McClure R, Lee H. VR23: A quinoline-sulfonyl hybrid proteasome inhibitor that selectively kills cancer via cyclin E-mediated centrosome amplification. Cancer Res. 2015;75(19):4164-75.
31. Song X, Zhang C, Zhao M, Chen H, Liu X, Chen J, et al. Steroid receptor coactivator-3 (SRC-3/AIB1) as a novel therapeutic target in triple-negative breast cancer and its inhibition with a phospho-bufalin prodrug. PLoS One. 2015;10(10)
32. Wei S, Cao H, Zhou X, Wu H, Yang J. Prokaryotically and eukaryotically expressed interleukin-24 induces breast cancer growth suppression via activation of apoptosis and inhibition of tumor angiogenesis. Mol Med Rep. 2015;11(5):3673-81.
33. Wu XY, Xu H, Wu ZF, Chen C, Liu JY, Wu GN, et al. Formononetin, a novel FGFR2 inhibitor, potently inhibits angiogenesis and tumor growth in preclinical models. Oncotarget. 2015;6(42):44563-78.
34. Yu Y, Cai W, Pei CG, Shao Y. Rhamnazin, a novel inhibitor of VEGFR2 signaling with potent antiangiogenic activity and antitumor efficacy. Biochem Biophys Res Commun. 2015;458(4):913-9.
35. Fouqué A, Delalande O, Jean M, Castellano R, Josselin E, Malleter M, et al. A novel covalent mTOR inhibitor, DHM25, shows in vivo antitumor activity against triple-negative breast cancer cells. J Med Chem. 2015;58(16):6559-73.
36. Marín-Ramos NI, Alonso D, Ortega-Gutiérrez S, Ortega-Nogales FJ, Balabasquer M, Vázquez-Villa H, et al. New inhibitors of angiogenesis with antitumor activity in vivo. J Med Chem. 2015;58(9):3757-66.
37. Ren XR, Wang J, Osada T, Mook RA Jr, Morse MA, Barak LS, et al. Perhexiline promotes HER3 ablation through receptor internalization and inhibits tumor growth. Breast Cancer Res. 2015;17(1):20.
38. Arpel A, Gamper C, Spenlé C, Fernandez A, Jacob L, Baumlin N, et al. Inhibition of primary breast tumor growth and metastasis using a neuropilin-1 transmembrane domain interfering peptide. Oncotarget. 2016;7(34):54723-32.
39. Brown WS, Tan L, Smith A, Gray NS, Wendt MK. Covalent targeting of fibroblast growth factor receptor inhibits metastatic breast cancer. Mol Cancer Ther. 2016;15(9):2096-106.
40. Fang J, Xiao L, Joo KI, Liu Y, Zhang C, Liu S, et al. A potent immunotoxin targeting fibroblast activation protein for treatment of breast cancer in mice. Int J Cancer. 2016;138(4):1013-23.
41. Park JS, Lee C, Kim HK, Kim D, Son JB, Ko E, et al. Suppression of the metastatic spread of breast cancer by DN10764 (AZD7762)-mediated inhibition of AXL signaling. Oncotarget. 2016;7(50):83308-18.
42. Prunier C, Josserand V, Vollaire J, Beerling E, Petropoulos C, Destaing O, et al. LIM kinase inhibitor Pyr1 reduces the growth and metastatic load of breast cancers. Cancer Res. 2016;76(12):3541-52.
43. Qin JJ, Wang W, Sarkar S, Voruganti S, Agarwal R, Zhang R. Inulalide A as a new dual inhibitor of NFAT1-MDM2 pathway for breast cancer therapy. Oncotarget. 2016;7(22):32566-78.
44. Shen F, Zhang Y, Jernigan DL, Feng X, Yan J, Garcia FU, et al. Novel small-molecule CX3CR1 antagonist impairs metastatic seeding and colonization of breast cancer cells. Mol Cancer Res. 2016;14(6):518-27.
45. Zhang J, Liu C, Shi W, Yang L, Zhang Q, Cui J, et al. The novel VEGF receptor 2 inhibitor YLL545 inhibits angiogenesis and growth in breast cancer. Oncotarget. 2016;7(27):41067-80.
46. Xu Z, Wang Z, Jia X, Wang L, Chen Z, Wang S, et al. MMGZ01, an anti-DLL4 monoclonal antibody, promotes nonfunctional vessels and inhibits breast tumor growth. Cancer Lett. 2016;372(1):118-27.
47. Zhou Q, Ji M, Zhou J, Jin J, Xue N, Chen J, et al. Poly (ADP-ribose) polymerase inhibitor, Zj6413, as a potential therapeutic agent against breast cancer. Biochem Pharmacol. 2016;107:29-40.
48. Gray MJ, Gong J, Hatch MM, Nguyen V, Hughes CC, Hutchins JT, et al. Phosphatidylserine-targeting antibodies augment the anti-tumorigenic activity of anti-PD-1 therapy by enhancing immune activation and downregulating pro-oncogenic factors induced by T-cell checkpoint inhibition in murine triple-negative breast cancers. Breast Cancer Res. 2016;18(1):50.
49. Chen WH, Song SS, Qi MH, Huan XJ, Wang YQ, Jiang H, et al. Discovery of potent 2,4-difluoro-linker poly(ADP-ribose) polymerase 1 inhibitors with enhanced water solubility and in vivo anticancer efficacy. Acta Pharmacol Sin. 2017;38(11):1521-32.
50. Dyari HRE, Rawling T, Chen Y, Sudarmano W, Bourget K, Dwyer JM, et al. A novel synthetic analogue of ω-3 17,18-epoxyeicosatetraenoic acid activates TNF receptor-1/ASK1/JNK signaling to promote apoptosis in human breast cancer cells. FASEB J. 2017;31(12):5246-57.
51. Humphries-Bickley T, Castillo-Pichardo L, Hernandez-O'Farrill E, Borrero-Garcia LD, Forestier-Roman I, Gero Y, et al. Characterization of a dual Rac/Cdc42 inhibitor MBQ-167 in metastatic cancer. Mol Cancer Ther. 2017;16(5):805-18.
52. Rathinavelu A, Alhazzani K, Dhandayuthapani S, Kanagasabai T. Anti-cancer effects of F16: A novel vascular endothelial growth factor receptor-specific inhibitor. Tumour Biol. 2017;39(11):1010428317726841.
53. Zhong P, Gu X, Cheng R, Deng C, Meng F, Zhong Z. αvβ3 integrin-targeted micellar mertansine prodrug effectively inhibits triple-negative breast cancer in vivo. Int J Nanomedicine. 2017;12:7913-21.
54. Li X, Yang C, Wan H, Zhang G, Feng J, Zhang L, et al. Discovery and development of pyrotinib: A novel irreversible EGFR/HER2 dual tyrosine kinase inhibitor with favorable safety profiles for the treatment of breast cancer. Eur J Pharm Sci. 2017;110:51-61.
55. Sugimoto Y, Sawant DB, Fisk HA, Mao L, Li C, Chettiar S, et al. Novel pyrrolopyrimidines as Mps1/TTK kinase inhibitors for breast cancer. Bioorg Med Chem. 2017;25(7):2156-66.
56. Torres-García D, Pérez-Torres A, Manoutcharian K, Orbe U, Servín-Blanco R, Fragoso G, et al. GK-1 peptide reduces tumor growth, decreases metastatic burden, and increases survival in a murine breast cancer model. Vaccine. 2017;35(42):5653-61.
57. Dominguez C, McCampbell KK, David JM, Palena C. Neutralization of IL-8 decreases tumor PMN-MDSCs and reduces mesenchymalization of claudin-low triple-negative breast cancer. JCI Insight. 2017;2(21)
58. Ebrahim HY, Akl MR, Elsayed HE, Hill RA, El Sayed KA. Usnic acid benzylidene analogues as potent mechanistic target of rapamycin inhibitors for the control of breast malignancies. J Nat Prod. 2017;80(4):932-52.
59. Huber-Ruano I, Raventós C, Cuartas I, Sánchez-Jaro C, Arias A, Parra JL, et al. An antisense oligonucleotide targeting TGF-β2 inhibits lung metastasis and induces CD86 expression in tumor-associated macrophages. Ann Oncol. 2017;28(9):2278-85.
60. Jahangir A, Chandra D, Quispe-Tintaya W, Singh M, Selvanesan BC, Gravekamp C. Immunotherapy with Listeria reduces metastatic breast cancer in young and old mice through different mechanisms. Oncoimmunology. 2017;6(9)
61. Riggs JR, Nagy M, Elsner J, Erdman P, Cashion D, Robinson D, et al. The discovery of a dual TTK protein kinase/CDC2-like kinase (CLK2) inhibitor for the treatment of triple-negative breast cancer initiated from a phenotypic screen. J Med Chem. 2017;60(21):8989-9002.
62. Yu W, Li C, Zhang W, Xia Y, Li S, Lin JY, et al. Discovery of an orally selective inhibitor of signal transducer and activator of transcription 3 using advanced multiple ligand simultaneous docking. J Med Chem. 2017;60(7):2718-31.
63. Alshaker H, Srivats S, Monteil D, Wang Q, Low CMR, Pchejetski D. Field template-based design and biological evaluation of new sphingosine kinase 1 inhibitors. Breast Cancer Res Treat. 2018;172(1):33-43.
64. Lee JJ, Kim HS, Lee JS, Park J, Shin SC, Song S, et al. Small molecule activator of NO23/NDPK as an inhibitor of metastasis. Sci Rep. 2018;8(1):10909.
65. Lei Q, Xiong L, Xia Y, Feng Z, Gao T, Wei W, et al. YLT-11, a novel PLK4 inhibitor, inhibits human breast cancer growth via inducing maladjusted centriole duplication and mitotic defect. Cell Death Dis. 2018;9(11):1066.
66. Li Y, Liu C, Tang K, Chen Y, Tian K, Feng Z, et al. Novel multi-kinase inhibitor T03 inhibits Taxol-resistant breast cancer. Mol Med Rep. 2018;17(2):2373-83.
67. Wang B, Shen J, Wang Z, Liu J, Ning Z, Hu M. Isomangiferin, a novel potent vascular endothelial growth factor receptor 2 kinase inhibitor, suppresses breast cancer growth, metastasis, and angiogenesis. J Breast Cancer. 2018;21(1):11-20.
68. Zhu D, Xu S, Deyanat-Yazdi G, Peng SX, Barnes LA, Norla RK, et al. Synthetic lethal strategy identifies a potent and selective TTK and CLK1/2 inhibitor for treatment of triple-negative breast cancer with a compromised G1-S checkpoint. Mol Cancer Ther. 2018;17(8):1727-38.
69. Chen Y, Ji M, Zhang S, Xue N, Xu H, Lin S, et al. Bt354 as a new STAT3 signaling pathway inhibitor against triple-negative breast cancer. J Drug Target. 2018;26(10):920-30.
70. Kasten BB, Oliver PG, Kim H, Fan J, Ferrone S, Zinn KR, et al. (212)Pb-labeled antibody 225.28 targeted to chondroitin sulfate proteoglycan 4 for triple-negative breast cancer therapy in mouse models. Int J Mol Sci. 2018;19(4):978.
71. Mariotto E, Viola G, Ronca R, Persano L, Aveic S, Bhujwalla ZM, et al. Choline kinase alpha inhibition by EB-3D triggers cellular senescence, reduces tumor growth, and metastatic dissemination in breast cancer. Cancers. 2018;10(10):405.
72. Capasso A, Bagby SM, Dailey KL, Currimjee N, Yacob BW, Ionkina A, et al. First-in-class phosphorylated-p68 inhibitor RX-5902 inhibits β-catenin signaling and demonstrates antitumor activity in triple-negative breast cancer. Mol Cancer Ther. 2019;18(11):1916-25.
73. Di L, Liu LJ, Yan YM, Fu R, Li Y, Xu Y, et al. Discovery of a natural small-molecule compound that suppresses tumor EMT, stemness, and metastasis by inhibiting TGFβ/BMP signaling in triple-negative breast cancer. J Exp Clin Cancer Res. 2019;38(1):134.
74. Garcia D, Nosarre P, Bonilla IV, Hilliard E, Peterson YK, Spruill L, et al. Development of a novel humanized monoclonal antibody to secreted frizzled-related protein-2 that inhibits triple-negative breast cancer and angiosarcoma growth in vivo. Ann Surg Oncol. 2019;26(13):4782-90.
75. Lee J, Lim B, Pearson T, Choi K, Fuson JA, Bartholomeusz C, et al. Anti-tumor and anti-metastasis efficacy of E6201, a MEK1 inhibitor, in preclinical models of triple-negative breast cancer. Breast Cancer Res Treat. 2019;175(2):339-51.
76. Park IH, Yang HN, Jeon SY, Hwang JA, Kim MK, Kong SY, et al. Anti-tumor activity of BET inhibitors in androgen-receptor-expressing triple-negative breast cancer. Sci Rep. 2019;9(1):13305.
77. Shen Y, Zhang W, Liu J, He J, Cao R, Chen X, et al. Therapeutic activity of DCC-2036, a novel tyrosine kinase inhibitor, against triple-negative breast cancer patient-derived xenografts by targeting AXL/MET. Int J Cancer. 2019;144(3):651-64.
78. Tian J, Chen X, Fu S, Zhang R, Pan L, Cao Y, et al. Bazedoxifene is a novel IL-6/GP130 inhibitor for treating triple-negative breast cancer. Breast Cancer Res Treat. 2019;175(3):553-66.
79. Viswanadhapalli S, Luo Y, Sareddy GR, Santhamma B, Zhou M, Li M, et al. EC359: A first-in-class small-molecule inhibitor for targeting oncogenic LIFR signaling in triple-negative breast cancer. Mol Cancer Ther. 2019;18(8):1341-54.
80. Zhou Z, Feng Z, Hu D, Yang P, Gur M, Bahar I, et al. A novel small-molecule antagonizes PRMT5-mediated KLF4 methylation for targeted therapy. EBioMedicine. 2019;44:98-111.
81. Cho TM, Kim JY, Kim YJ, Sung D, Oh E, Jang S, et al. C-terminal HSP90 inhibitor L80 elicits anti-metastatic effects in triple-negative breast cancer via STAT3 inhibition. Cancer Lett. 2019;447:141-53.
82. Idrissou M, Judes G, Daures M, Sanchez A, El Ouardi D, Besse S, et al. TIP60 inhibitor TH1834 reduces breast cancer progression in xenografts in mice. Omics. 2019;23(9):457-9.
83. Kawai J, Toki T, Ota M, Inoue H, Takata Y, Asahi T, et al. Discovery of a potent, selective, and orally available MTHFD2 inhibitor (DS18561882) with in vivo antitumor activity. J Med Chem. 2019;62(22):10204-20.
84. Kim KY, Yoon M, Cho Y, Lee KH, Park S, Lee SR, et al. Targeting metastatic breast cancer with peptide epitopes derived from the autocatalytic loop of Prss14/ST14 membrane serine protease and with monoclonal antibodies. J Exp Clin Cancer Res. 2019;38(1):404.
85. Qiao Z, Li X, Kang N, Yang Y, Chen C, Wu T, et al. A novel specific anti-CD73 antibody inhibits triple-negative breast cancer cell motility by regulating autophagy. Int J Mol Sci. 2019;20(5):1050.
86. Xiao H, Claret FX, Shen Q. The novel Jab1 inhibitor CSN5i-3 suppresses cell proliferation and induces apoptosis in human breast cancer cells. Neoplasma. 2019;66(3):481-6.
87. Deng S, Krutilin RI, Wang Q, Lin Z, Parke DN, Playa HC, et al. An orally available tubulin inhibitor, VERU-111, suppresses triple-negative breast cancer tumor growth and metastasis and bypasses taxane resistance. Mol Cancer Ther. 2020;19(2):348-63.
88. Hu P, Shang L, Chen J, Chen X, Chen C, Hong W, et al. A nanometer-sized protease inhibitor for precise cancer diagnosis and treatment. J Mater Chem B. 2020;8(3):504-14.
89. Kennedy SP, O'Neill M, Cunningham D, Morris PG, Toomey S, Blanco-Aparicio C, et al. Preclinical evaluation of a novel triple-acting PIM/PI3K/mTOR inhibitor, IBL-302, in breast cancer. Oncogene. 2020;39(14):3028-40.
90. Pan L, Chen X, Fu S, Yu W, Li C, Wang T, et al. LLY17, a novel small molecule STAT3 inhibitor, induces apoptosis and suppresses cell migration and tumor growth in triple-negative breast cancer. Breast Cancer Res Treat. 2020;181(1):31-41.
91. Lin L, Luo X, Wang L, Xu F, He Y, Wang Q, et al. BML-111 inhibits EMT, migration, and metastasis of TAMs-stimulated triple-negative breast cancer cells via the ILK pathway. Int Immunopharmacol. 2020;85:106625.
92. Liu JH, Chen C, Li ZY, Zou ZM, Gao DC, Zhang X, et al. The MyD88 inhibitor TJ-M2010-2 suppresses proliferation, migration, and invasion of breast cancer cells by regulating MyD88/GSK-3β and MyD88/NF-κB signaling pathways. Exp Cell Res. 2020;394(2):112157.
93. Wu R, Yu W, Yao C, Liang Z, Yoon Y, Xie Y, et al. Amide-sulfamide modulators as effective anti-tumor metastatic agents targeting the CXCR4/CXCL12 axis. Eur J Med Chem. 2020;185:111823.
94. Chen J, Li N, Liu B, Ling J, Yang W, Pang X, et al. Pracinostat (SB939), a histone deacetylase inhibitor, suppresses breast cancer metastasis and growth by inactivating the IL-6/STAT3 signaling pathways. Life Sci. 2020;248:117469.
95. Andrini L, Marin GH, Inda AM, Bruzzoni-Giovanelli H, Garcia M, Errecalde J, et al. Anti-tumoral effect of a cell-penetrating and interfering peptide targeting PP2A/SET interaction. Folia Med (Plovdiv). 2020;62(1):31-6.
96. Li Q, Cao J, He Y, Liu X, Mao G, Wei B, et al. R5, a neutralizing antibody to Robo1, suppresses breast cancer growth and metastasis by inhibiting angiogenesis via down-regulating filamin A. Exp Cell Res. 2020;387(1):111756.
97. Mahmood N, Arakelian A, Khan HA, Tanvir I, Mazar AP, Rabbani SA. uPAR antibody (huATN-658) and Zometa reduce breast cancer growth and skeletal lesions. Bone Res. 2020;8(1):30.
98. Park SK, Byun WS, Lee S, Han YT, Jeong YS, Jang K, et al. A novel small molecule STAT3 inhibitor SLSI-1216 suppresses proliferation and tumor growth of triple-negative breast cancer cells through apoptotic induction. Biochem Pharmacol. 2020;178:114053.
99. van de Merbel AF, van Hooij O, van der Horst G, van Rijt-van de Westerlo CCM, van der Mark MH, Cheung H, et al. Identification of small molecule inhibitors that reduce invasion and metastasis of aggressive cancers. Int J Mol Sci. 2021;22(4):1422.
100. Zhao R, Fu L, Yuan Z, Liu Y, Zhang K, Chen Y, et al. Discovery of a novel small-molecule inhibitor of Fam20C that induces apoptosis and inhibits migration in triple-negative breast cancer. Eur J Med Chem. 2021;210:113088.
101. Li Y, Yang G, Zhang J, Tang P, Yang C, Wang G, et al. Discovery, synthesis, and evaluation of a highly selective vascular endothelial growth factor receptor 3 (VEGFR3) inhibitor for the potential treatment of metastatic triple-negative breast cancer. J Med Chem. 2021;64(16):12022-48.
102. Yi M, Wu Y, Niu M, Zhu S, Zhang J, Yan Y, et al. Anti-TGF-β/PD-L1 bispecific antibody promotes T cell infiltration and exhibits enhanced antitumor activity in triple-negative breast cancer. J Immunother Cancer. 2022;10(12)
103. Zhang Y, Chen J, Mi D, Ling J, Li H, He P, et al. Discovery of YH677 as a cancer stemness inhibitor that suppresses triple-negative breast cancer growth and metastasis by regulating the TGFβ signaling pathway. Cancer Lett. 2023;560:216142.
104. Dai X, Hou Y, Deng T, Lin G, Cao Y, Yu G, et al. A specific RAGE-binding peptide inhibits triple-negative breast cancer growth through blocking of Erk1/2/NF-κB pathway. Eur J Pharmacol. 2023;954:175861.
105. Gao G, Li J, Cao Y, Li X, Qian Y, Wang X, et al. Design, synthesis, and biological evaluation of novel 4,4'-bipyridine derivatives acting as CDK9-Cyclin T1 protein-protein interaction inhibitors against triple-negative breast cancer. Eur J Med Chem. 2023;261:115858.
106. Ali N, Wolf C, Kanchan S, Veerabhadraiah SR, Bond L, Turner MW, et al. 9S1R nullomer peptide induces mitochondrial pathology, metabolic suppression, and enhanced immune cell infiltration in a triple-negative breast cancer mouse model. Biomed Pharmacother. 2024;170:115997.
107. Desroys du Roure P, Lajoie L, Mallavialle A, Alcaraz LB, Mansouri H, Fenou L, et al. A novel Fc-engineered cathepsin D-targeting antibody enhances ADCC, triggers tumor-infiltrating NK cell recruitment, and improves treatment with paclitaxel and enzalutamide in triple-negative breast cancer. J Immunother Cancer. 2024;12(1)
108. Lyu L, Li H, Lu K, Jiang S, Li H. PAK inhibitor FRAX486 decreases the metastatic potential of triple-negative breast cancer cells by blocking autophagy. Br J Cancer. 2024;130(3):394-405.

**S1**
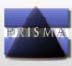
**1 File. PRISMA 2020 Checklist.**

| **Section and Topic** | **Item #** | **Checklist item** | **Location where item is reported** |
| --- | --- | --- | --- |
| **TITLE** | | |  |
| Title | 1 | Identify the report as a systematic review. | Title lines 1- 2 |
| **ABSTRACT** | | |  |
| Abstract | 2 | See the PRISMA 2020 for Abstracts checklist. | Lines 35-56 |
| **INTRODUCTION** | | |  |
| Rationale | 3 | Describe the rationale for the review in the context of existing knowledge. | Lines 60-106 |
| Objectives | 4 | Provide an explicit statement of the objective(s) or question(s) the review addresses. | Lines 106-110 |
| **METHODS** | | |  |
| Eligibility criteria | 5 | Specify the inclusion and exclusion criteria for the review and how studies were grouped for the syntheses. | Supplementary file-Table 1 and Lines 144-159 in manuscript |
| Information sources | 6 | Specify all databases, registers, websites, organisations, reference lists and other sources searched or consulted to identify studies. Specify the date when each source was last searched or consulted. | Lines 125-142 |
| Search strategy | 7 | Present the full search strategies for all databases, registers and websites, including any filters and limits used. | Supplementary file- search strategy and Lines 125-142 in manuscript |
| Selection process | 8 | Specify the methods used to decide whether a study met the inclusion criteria of the review, including how many reviewers screened each record and each report retrieved, whether they worked independently, and if applicable, details of automation tools used in the process. | Lines 145-159 |
| Data collection process | 9 | Specify the methods used to collect data from reports, including how many reviewers collected data from each report, whether they worked independently, any processes for obtaining or confirming data from study investigators, and if applicable, details of automation tools used in the process. | Lines 160-183 |
| Data items | 10a | List and define all outcomes for which data were sought. Specify whether all results that were compatible with each outcome domain in each study were sought (e.g. for all measures, time points, analyses), and if not, the methods used to decide which results to collect. | Lines 197-230 |
|  | 10b | List and define all other variables for which data were sought (e.g. participant and intervention characteristics, funding sources). Describe any assumptions made about any missing or unclear information. | Lines 197-230 |
| Study risk of bias assessment | 11 | Specify the methods used to assess risk of bias in the included studies, including details of the tool(s) used, how many reviewers assessed each study and whether they worked independently, and if applicable, details of automation tools used in the process. | Not applicable |
| Effect measures | 12 | Specify for each outcome the effect measure(s) (e.g. risk ratio, mean difference) used in the synthesis or presentation of results. | Lines197-230 |
| Synthesis methods | 13a | Describe the processes used to decide which studies were eligible for each synthesis (e.g. tabulating the study intervention characteristics and comparing against the planned groups for each synthesis (item #5)). | Lines 197-230 |
|  | 13b | Describe any methods required to prepare the data for presentation or synthesis, such as handling of missing summary statistics, or data conversions. | Lines 197-230 |
|  | 13c | Describe any methods used to tabulate or visually display results of individual studies and syntheses. | Lines 197-230 |
|  | 13d | Describe any methods used to synthesize results and provide a rationale for the choice(s). If meta-analysis was performed, describe the model(s), method(s) to identify the presence and extent of statistical heterogeneity, and software package(s) used. | Lines 197-230 |
|  | 13e | Describe any methods used to explore possible causes of heterogeneity among study results (e.g. subgroup analysis, meta-regression). | Lines 197-230 |
|  | 13f | Describe any sensitivity analyses conducted to assess robustness of the synthesized results. | Not applicable |
| Reporting bias assessment | 14 | Describe any methods used to assess risk of bias due to missing results in a synthesis (arising from reporting biases). | Not applicable |
| Certainty assessment | 15 | Describe any methods used to assess certainty (or confidence) in the body of evidence for an outcome. | Not applicable |
| **RESULTS** | | |  |
| Study selection | 16a | Describe the results of the search and selection process, from the number of records identified in the search to the number of studies included in the review, ideally using a flow diagram. | Lines 236-252 |
|  | 16b | Cite studies that might appear to meet the inclusion criteria, but which were excluded, and explain why they were excluded. | Figure 1 |
| Study characteristics | 17 | Cite each included study and present its characteristics. | Supplementary file- tables 3,4,5,6, and references |
| Risk of bias in studies | 18 | Present assessments of risk of bias for each included study. | Not applicable |
| Results of individual studies | 19 | For all outcomes, present, for each study: (a) summary statistics for each group (where appropriate) and (b) an effect estimate and its precision (e.g. confidence/credible interval), ideally using structured tables or plots. | Figures 3 and 5 result part |
| Results of syntheses | 20a | For each synthesis, briefly summarise the characteristics and risk of bias among contributing studies. | Supplementary file table 7 |
|  | 20b | Present results of all statistical syntheses conducted. If meta-analysis was done, present for each the summary estimate and its precision (e.g. confidence/credible interval) and measures of statistical heterogeneity. If comparing groups, describe the direction of the effect. | Result part |
|  | 20c | Present results of all investigations of possible causes of heterogeneity among study results. | Figures 4, 6 result part |
|  | 20d | Present results of all sensitivity analyses conducted to assess the robustness of the synthesized results. | Not applicable |
| Reporting biases | 21 | Present assessments of risk of bias due to missing results (arising from reporting biases) for each synthesis assessed. | Figure 7 and supplementary file figure 1 |
| Certainty of evidence | 22 | Present assessments of certainty (or confidence) in the body of evidence for each outcome assessed. | Not applicable |
| **DISCUSSION** | | |  |
| Discussion | 23a | Provide a general interpretation of the results in the context of other evidence. | Lines 464-511 |
|  | 23b | Discuss any limitations of the evidence included in the review. | Lines 512-537 |
|  | 23c | Discuss any limitations of the review processes used. | Lines 512-537 |
|  | 23d | Discuss implications of the results for practice, policy, and future research. | Lines 512-537 |
| **OTHER INFORMATION** | | |  |
| Registration and protocol | 24a | Provide registration information for the review, including register name and registration number, or state that the review was not registered. | Lines 112-119 |
|  | 24b | Indicate where the review protocol can be accessed, or state that a protocol was not prepared. | Lines 112-119 |
|  | 24c | Describe and explain any amendments to information provided at registration or in the protocol. | Lines 119-124 |
| Support | 25 | Describe sources of financial or non-financial support for the review, and the role of the funders or sponsors in the review. | See Funding section |
| Competing interests | 26 | Declare any competing interests of review authors. | There are no conflicts of interest to declare |
| Availability of data, code and other materials | 27 | Report which of the following are publicly available and where they can be found: template data collection forms; data extracted from included studies; data used for all analyses; analytic code; any other materials used in the review. | The data and analytic code for this study are available on the Open Science Framework  See Link at Data availability section |

*From:*  Page MJ, McKenzie JE, Bossuyt PM, Boutron I, Hoffmann TC, Mulrow CD, et al. The PRISMA 2020 statement: an updated guideline for reporting systematic reviews. BMJ 2021;372:n71. doi: 10.1136/bmj.n71

For more information, visit: <http://www.prisma-statement.org/>
